# Exploring Spatio-temporal Dynamics of Cellular Automata for Pattern Recognition in Networks

**DOI:** 10.1038/srep37329

**Published:** 2016-11-22

**Authors:** Gisele Helena Barboni Miranda, Jeaneth Machicao, Odemir Martinez Bruno

**Affiliations:** 1 Institute of Mathematics and Computer Science, University of São Paulo, São Carlos - SP, Brazil; 2 São Carlos Institute of Physics, University of São Paulo, São Carlos - SP, PO Box 369, 13560-970, Brazil

## Abstract

Network science is an interdisciplinary field which provides an integrative approach for the study of complex systems. In recent years, network modeling has been used for the study of emergent phenomena in many real-world applications. Pattern recognition in networks has been drawing attention to the importance of network characterization, which may lead to understanding the topological properties that are related to the network model. In this paper, the Life-Like Network Automata (LLNA) method is introduced, which was designed for pattern recognition in networks. LLNA uses the network topology as a tessellation of Cellular Automata (CA), whose dynamics produces a spatio-temporal pattern used to extract the feature vector for network characterization. The method was evaluated using synthetic and real-world networks. In the latter, three pattern recognition applications were used: (i) identifying organisms from distinct domains of life through their metabolic networks, (ii) identifying online social networks and (iii) classifying stomata distribution patterns varying according to different lighting conditions. LLNA was compared to structural measurements and surpasses them in real-world applications, achieving improvement in the classification rate as high as 23%, 4% and 7% respectively. Therefore, the proposed method is a good choice for pattern recognition applications using networks and demonstrates potential for general applicability.

Networks have been successfully used in many areas of knowledge that covers practically all fields of Science: Earth^1^,^2^,^3^,^4^,^5^,^6^, Social^7^,^8^,^9^,^10^,^11^,^12^, Life^13^,^14^,^15^,^16^,^17^,^18^, Physical^19^,^20^,^21^,^22^,^23^ and Formal Sciences^24^,^25^,^26^,^27^. The main reason behind the growing interest in networks lies in the fact that it shows a different perspective of the traditional data analysis. During centuries, the scientific research paradigm was ruled by the reductionist approach. Scientific and technological advances increased the amount of data and also encouraged the development of powerful computers, which are capable of processing and storing this huge amount of data. This scenario, often called “big data”^28^, requires the development of an integrative paradigm of science. Complex systems, in particular, chaos theory and networks are research fields that have contributed with interesting approaches to this scenario. Both have shown to be able to handle multiple actors, multiple events and multiple variable problems^29^,^30^,^31^. Particularly, networks are a good approach to model complex systems once they incorporate the connectivity among the elements of the system.

During the last decades, Pattern Recognition (PR) has been widely used in both fundamental and applied sciences. Remarkably, most of the PR applications deals with a big amount of data which are difficult handle with the reductionist approach. A classical example is the medical field, where computational and mathematical methods dealing with huge amount of data allowed a strong innovation in the field. Networks are a natural tool for data modeling. In face of that, the combination of PR and networks arises as an important alternative in the big data scenario for finding, identifying, analyzing, and clustering patterns that are unfeasible to deal with other approaches. Pattern recognition in networks aims at the characterization of networks by extracting information regarding the correlation between vertices and their relationship with topology. This information may lead to the comprehension of network patterns that are intrinsically related to the network model. Therefore, the choice of adequate network descriptors is crucial for this kind of applications. Many measurements can be extracted from the network topology and be used to distinguish network types^32^. These measurements can be related to connectivity attributes, such as the mean degree and the degree distributions and correlations. Distances and path lengths are also important topological attributes when the spatial position of nodes is relevant. Moreover, there are measurements related to cycles in networks such as transitivity and the clustering coefficient^33^, which quantifies the small-world phenomenon in networks. We can also mention centrality measures, such as betweenness, closeness and eigenvectors. Other measurements include spectral and hierarchical measures as well as fractal dimension among many examples^32^.

Structural measures have been investigated mainly in the context of network analysis, however much less effort was made in pattern recognition applications. A few related studies have addressed this challenging topic and have had significant advances. Costa *et al*.^34^ analyzed both traditional measures regarding structural properties of networks and methods for dimensionality reduction, as many measures can be correlated to each other. They also discuss the possibility of expanding classical pattern recognition techniques to network analysis. Moreover, Golçalves *et al*.^35^ proposed a method based on partially self-avoiding deterministic walks to classify network models using the agent trajectory over the network topology. The joint distribution of the transient time and the cycle period were used to compose the feature vector. Their results indicate an improvement in the correct classification rate when compared to traditional network measures. Networks have also been used to perform pattern recognition tasks in Computer Vision, such as contour^36^,^37^ and texture analysis^38^,^39^.

In this paper we proposed the Life-Like Network Automata (LLNA) which was designed as a method for network analysis for pattern recognition applications. In the LLNA approach, networks are modeled as the CA’s tessellation and the spatio-temporal pattern obtained from the evolution of the CA is used to extract the feature vector for network characterization. Life-Like Network Automata uses a family of CAs inspired by the rules of Life-like, which is an extension of the popular Conway’s Game of Life^40^. The network descriptor is obtained from the spatio-temporal pattern as described in Fig. 1.

Cellular Automata (CA) are dynamical systems defined over tessellations of the Euclidean space, which are governed by deterministic rules that define the states of the cells at each time step. CAs are essentially discrete, *i*.*e*., time, space and the set of states are discrete. In recent years, CAs were largely explored as modeling tools of systems characterized by many variables which would be difficult to handle with partial differential equations. On the other hand, the evolved spatio-temporal patterns can provide emergent behavior, resulting from the dynamics of each individual cell. Therefore, they have also become a relevant tool for the study of complexity and the formation of spatio-temporal patterns^41^. CA were originally designed in regular tessellations (square-grids), notwithstanding, most of the real-world systems are built upon irregular tessellations and present topologies that are much more complex such as the scale-free networks.

In the 1990s, studies modeling CAs on irregular tessellations began appearing in the literature. The first studies integrating both areas of Networks and CAs can be found in refs 42, 43. Watts discusses CA computation in small-world networks in tasks, such as the density classification problem and synchronization. Tomassini *et al*. discuss properties of small-world networks in the global computing capacity of CA, such as the robustness of the network topology^44^. Marr & Hütt^45^,^46^ also studied the dynamics of evolving networks through the use of CAs. Their results indicate a strong association between entropy measurements obtained from the spatio-temporal patterns and the degree distribution of a network. Moreover, the majority problem and some related rules are explored in this context. Dynamic pattern evolution was also studied by Zhou & Lipowsky^47^ regarding scale-free topology. The Ising model is used to describe the states of each vertex that evolve according to local majority rules. The authors found that scale-free networks present qualitatively different dynamic behavior given a threshold exponent of *γ*/2 (*γ* is the power law exponent). In other related works, the network topology was also explored using CAs and other dynamical models^48^,^49^,^50^.

In contrast, LLNA is based on the spatio-temporal patterns of a binary CA governed by the dynamics of rules inspired by Life-like CA. Instead of using the number of living cells, the proposed CA performs a mapping between the density of living neighbors and a specific Life-like rule. We evaluated LLNA in two distinct types of applications: synthetic networks and real-world networks. In the former, we performed the classification of theoretical network models in two experiments: general and scale-free models. We used well-known general models namely, random, small-world, scale-free and geographical. For the scale-free classification, we considered five categories of scale-free networks generated according to the models proposed by Barabási & Albert^51^ and Dorogovtsev & Mendes^52^. In the latter, we performed classification tasks for real-world applications that use networks as data representation. These data are composed by samples of different categories, and therefore, their automatic identification remains an important problem for each specific application. We used LLNA in three pattern recognition applications: (i) identifying organisms from distinct domains of life, *Archaea, Bacteria* and *Eukaryota*, through their metabolic networks. The dataset used was first studied by Jeong *et al*.^13^; (ii) identifying structural patterns in two online social networks, *Twitter* and *Google*+, using the samples of social interactions obtained from the SNAP database^53^, and, finally, (iii) classifying stomata distribution patterns varying according to different lighting conditions^54^. Using theoretical network models supports the understanding of the obtained results, as these topologies present known properties, and using real-world networks is a strong evidence that LLNA is a good choice for pattern recognition applications using networks and demonstrates general applicability.

## Results

### Life-Like Network Automata (LLNA)

LLNA is a method for pattern recognition which uses a family of CAs inspired by the rules of Life-like. The choice of the Life-like family was due to the flexibility of these CAs which provide a vast rule space^55^,^56^,^57^. CAs are usually represented on regular tessellations (square grids) in n-dimensional Euclidean spaces, 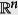, and the set of transition rules, Φ, is defined over a fixed number of neighbors. However, when considering CAs built upon irregular tessellations^45^,^58^,^59^, such as networks, the number of neighbors of each cell may vary considerably. This issue can restrict the comparison between two systems. To overcome this, we focused on a particular solution^42^,^45^ that uses the neighborhood density instead of the number of neighbors alive when applying the transition rules.

Given a CA described by the quintuple 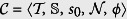, we assume the following correspondences: (i) the tessellation 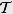 is represented by the network. In this approach, every network node is considered as a CA cell, *i*.*e*. both terms “node” and “cell” are used here interchangeably. (ii) The set of states 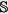 is composed by two elements, such that 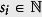, where *s*_*i*_ = 0 represents the “dead” state and *s*_*i*_ = 1 represents the “alive” state. (iii) *s*_0_ is the initial configuration of the states for all the cells 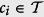 *i*.*e*., *s*(*c*_*i*_, 0) = *s*_0_(*c*_*i*_). (iv) The set of neighbors 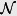 is given by the adjacency matrix *A*, where *A*_*ij*_ = 1, if *i* is connected to *j* and *A*_*ij*_ = 0, otherwise. Thus, the number of neighbors or degree of node *i* is defined as: 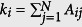, where *N* is the total number of nodes. As expected, *k*_*i*_ varies for each node and, therefore, each type of network has a characteristic degree distribution. Moreover, the neighborhood density (*ρ*_*i*_) of node *i* for a given state *s*_*k*_ = *s* can be generically defined by


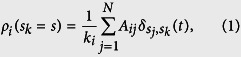


where 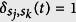, if *s*_*j*_ = *s*_*k*_ and 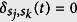, otherwise. Specifically, *A*_*ij*_ defines the neighborhood relation and 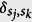 is the condition that the nodes hold the same state. For instance, for a binary state space, *ρ*_*i*_ can be simplified as 
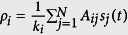
. Finally, the last correspondence (v) is given by the transition function *ϕ*, which determines the state of cell *c*_*i*_ in time *t*. The classic Life-like CA can be characterized by the notation B*x*/S*y* (e.g. B23/S3, B18/S246, B567/S09, etc), such that, 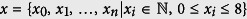 and 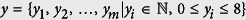 are two sets corresponding to the numbers of possible living cells that satisfy the conditions of birth and survival. Notice that, when combining these conditions, there is a total of 2^(9+9)^ (=262144) Life-like rules. This family of CAs are defined over a two dimensional regular tessellation and their neighbors are given by Moore’s neighborhood which is composed by the eight nearest neighbors. Therefore, B and S are sets containing from zero up to eight elements (additional information about Life-like CAs can be found in section S1 of supplementary material). We traced a correspondence between the number of alive neighbors, given by the Life-like rule, and the density *ρ*_*i*_ of each network node. This correspondence takes place with the definition of nine intervals. The first eight intervals are defined as 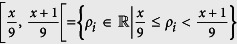, where *x* is defined by the value of the Life-like rule, and, the last interval as 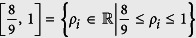. The same intervals are defined similarly for *y*. The function


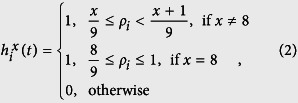


verifies whether the interval defined over *x* is satisfied for node *i*. For instance, considering rule B3/S23, three neighbors must be alive so that a node is born (*x* = 3), therefore the birth condition is given by: 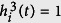, while the survival condition is given by 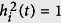 or 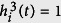. Finally, the transition function for LLNA is defined by


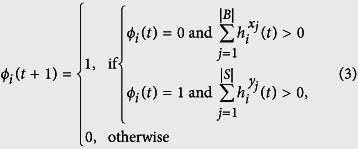


where *ϕ*_*i*_(*t* + 1) will be the state of node *i* in the next time step and *ϕ*_*i*_(*t*) is its current state.

**Figure 2:**
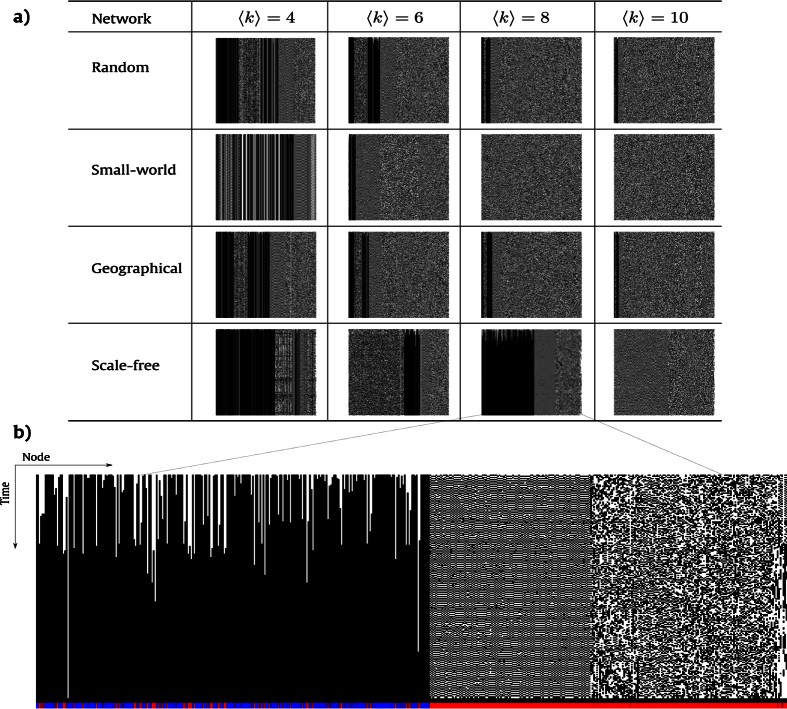
Space-time diagrams for different network models: random, small-world, scale-free and geographical. These networks were evolved using rule B1357/S2468. (**a**) All the diagrams of this figure were obtained for networks generated with *N* = 500 nodes and four different values of 〈*k*〉. The CA was evolved for *t* = 500 time steps. (**b**) Highlighted space-time diagram of a scale-free network with 〈*k*〉 = 8. The states of the nodes are represented horizontally (from left to right), where the white pixels correspond to the “alive” nodes and the black pixels to the “dead” nodes. Each time step *t* is represented vertically. The colors observed at the bottom of the diagram correspond to the values of entropy of each node. The red cells correspond to the highest entropy values while the blue cells, to the lowest.

Figure 2(a) shows the spatio-temporal diagrams obtained for random, small-world, scale-free and geographical networks which were evolved by rule B1357/S2468 according to Eq. 3. All the networks used to obtain the respective diagrams present *N* = 500 nodes and different mean degree 〈*k*〉 and they were evolved for *t* = 500 time steps. Initially, in *t* = 0, a possible state is assigned to each node according to a uniform distribution. The space-time diagram depicts the pattern formation where each column represents a node while each row represents the time evolution of the states for each node. Traditionally, for elementary CAs, every node is surrounded by its neighbors, since the number of neighbors is fixed. However, in the diagrams of Fig. 2, the neighborhood relation was not preserved due to variations in the degree of the nodes. Nevertheless, the columns were ordered according to their connectivity where the left-most corresponds to nodes with the smallest values of *k*_*i*_, and, the right-most, to the ones with the largest values of *k*_*i*_.

There are four main patterns observed in these diagrams in terms of dynamics: stable, oscillating, chaotic and complex. For instance, all of these patterns can be observed in different regions of the highlighted diagram of Fig. 2(b), which was obtained using a scale-free network as tessellation with 〈*k*〉 = 8. The colors at the bottom of this figure are related to values of the Shannon entropy which quantifies how homogeneous is the evolution pattern and is defined in the Materials and Methods section. Red cells represent the highest entropy values. For this example network, hubs tend to present chaotic and complex patterns once they are on the right-most side of this diagram. Whereas in the diagram of the same network model with 〈*k*〉 = 10 there are no stable patterns. Moreover, the spatio-temporal diagrams of the other models also present some of these patterns, although they may change considerably regarding the area of occupation. Random patterns appear more frequently as 〈*k*〉 increases, consequently, the entropy also increases. This effect is due to the addition of new edges in the network.

**Figure 3:**
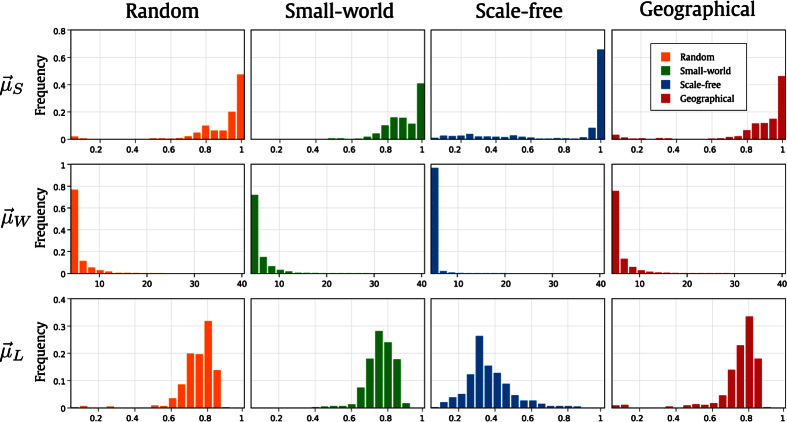
Histogram of the three distributions used to quantitatively analyze the spatio-temporal patterns of distinct network models: Shannon entropy 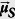, word length 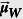 and Lempel-Ziv complexity 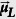. The following parameters were adopted: *N* = 500, 〈*k*〉 = 4 and *t* = 350.

The Shannon entropy, the word length and the Lempel-Ziv complexity were used to assess the spatio-temporal patterns (see section S2 of supplementary material for details about their definitions). We have investigated how these measurements vary for the different topologies studied in this paper. The evolution provided by each network node was analyzed in terms of a time series containing only zeros and ones. Except for the word length, which is calculated for the whole diagram, and, therefore is a global measurement, the other two, the Shannon entropy and the Lempel-Ziv complexity, were calculated for each network node. Then, the distributions of the three measurements were obtained and the corresponding histograms are illustrated in Fig. 3. Each row represents a distribution: Shannon entropy 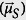, word length frequency 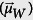 and the Lempel-Ziv complexity 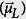, while each column represents a different network model. The networks used to generate these histograms present *N* = 500, 〈*k*〉 = 4 and *t* = 350. It can be observed that the scale-free network at Fig. 3 presents large frequency of nodes with high entropy by comparing 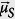 among the different network topologies. These nodes correspond to the nodes with the highest values of 〈*k*〉, as observed in Fig. 2. The histogram corresponding to the other topologies also present large frequency of nodes with high entropy. This is due to the presence of oscillating spatio-temporal patterns. Regarding 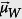, the respective histograms show that the most frequent words are the smallest ones. Moreover, the Lempel-Ziv distribution 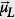 also shows significant differences among the network models.

### Analysis and selection of parameters

**Figure 4:**
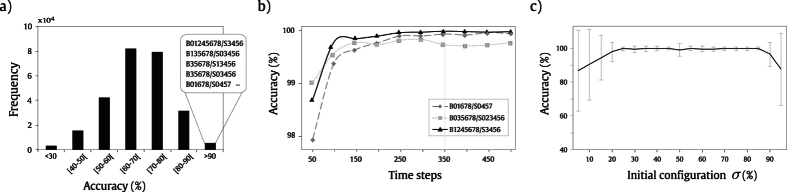
(**a**) Accuracy distribution for the 262144 rules of the Life-like family regarding the correct classification rate of network models (random, small-world, scale-free and geographical). The highlighted rules provided the best results. (**b**) Accuracy (%) in relation to the evolved time *t* for the three highlighted rules. (**c**) Accuracy (%) for different initial distributions of alive nodes (σ) using rule B135678/S03456.

LLNA can be influenced by the following parameters: the Life-like rule; *t*, the number of evolution steps of the automaton, and, σ, the percentage of the initial alive population in *t* = 0. The selection of the Life-like rule for a pattern recognition application is performed through an optimization procedure in which classification accuracy is maximized. In this context, accuracy is the percentage of correct classified instances. All Life-like rules are evaluated regarding the accuracy they provide as transition function (see Eq. 3) of the proposed Life-Like Network Automata. We have conducted an experiment in order to find the most discriminating rules for classifying network models using this optimization procedure. Therefore, each network model was defined as a class in this experiment: random, small-world, scale-free and geographical. We used the *rule*-*selection*-*dataset* which contains networks of each theoretical model and is described in detail in the Materials and Methods section. Figure 4(a) presents the histogram of the accuracy achieved by all Life-like rules. We used k-NN classifier and the Shannon entropy distribution 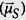 as feature vector. It can be observed that the majority of the rules provided accuracies greater than 60% and a set of specific rules provided accuracies greater than 90%. From this set, we selected the 10 rules which provided the highest accuracies in order to be used in the subsequent experiments with synthetic networks.

We also analyzed whether accuracy may be affected by the other two parameters: *t* and σ and the results are shown in Fig. 4(b) and (c), respectively. In the first one, we can observe that the correct classification rate tends to increase as the values of *t* also increases. The initial accuracy is already high given that the three illustrative rules are amongst the ten previously selected rules. There is also a rapid convergence of the accuracy values, which was observed for the three analyzed rules, although this behavior cannot be assumed for all the rules. However, for patterns that do not converge, an increase in the number of time steps may provide more details about the topology being evolved. Regarding the influence of the number of alive nodes in the initial configuration of the automaton, we have σ representing the probability of having cells *c*_*i*_ such that *s*(*c*_*i*_) = 1 at *t* = 0. We performed the same experiment of network classification considering different values of σ. We observed that values of σ close to a uniform distribution of states, *i*.*e*. σ = 50%, provided the highest accuracies, as shown in Fig. 4(c).

Based on the observed behavior of *t* and σ, we adopted the following values *t* = 350 and σ = 50% in the subsequent experiments performed in this paper. Additionally, we performed an analysis of the influence of the number of network nodes, *N*, which is presented in Section S5 of supplementary material.

### Pattern recognition in synthetic networks

This section presents three experiments with synthetic networks in order to illustrate the pattern recognition approach of Life-Like Network Automata and also to validate the parameters obtained in the training phase, as shown in the previous section. Similarly to the training phase, the first experiment also aims at the classification of network models (random, small-world, scale-free and geographical). However, a new dataset, named *synthetic*-*dataset*, was generated containing other samples of the same network models. Therefore, there is no intersection between the *rule*-*selection*-*dataset* and the *synthetic*-*dataset* (see Materials and Methods section for a complete description of both datasets). The networks present different combinations of *N* and 〈*k*〉 in order to increase the heterogeneity of *synthetic*-*dataset*. We compared the performance of LLNA with the following structural measurements of networks: average degree (〈*k*〉), average hierarchical degree of level 1 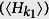, average hierarchical degree of level 2 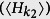, average clustering coefficient (〈*cc*〉), average path length (*l*) and degree Pearson correlation (*ρ*_*P*_). For LLNA, we used the following measurements extracted from the spatio-temporal patterns: Shannon entropy, word length and Lempel-Ziv complexity. The distribution of these measurements 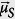, 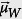 and 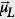, were used as feature vectors, respectively, as well as the combination of them 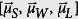. We also tested the accuracy of the average values of those measurements as feature vectors: [〈*μ*_*S*_〉, 〈*μ*_*W*_〉, 〈*μ*_*L*_〉]. The structural measurements were also evaluated both individually and combined. All the experiments presented in this section were performed using SVM classifier and 10-fold cross validation.

**Figure 5:**
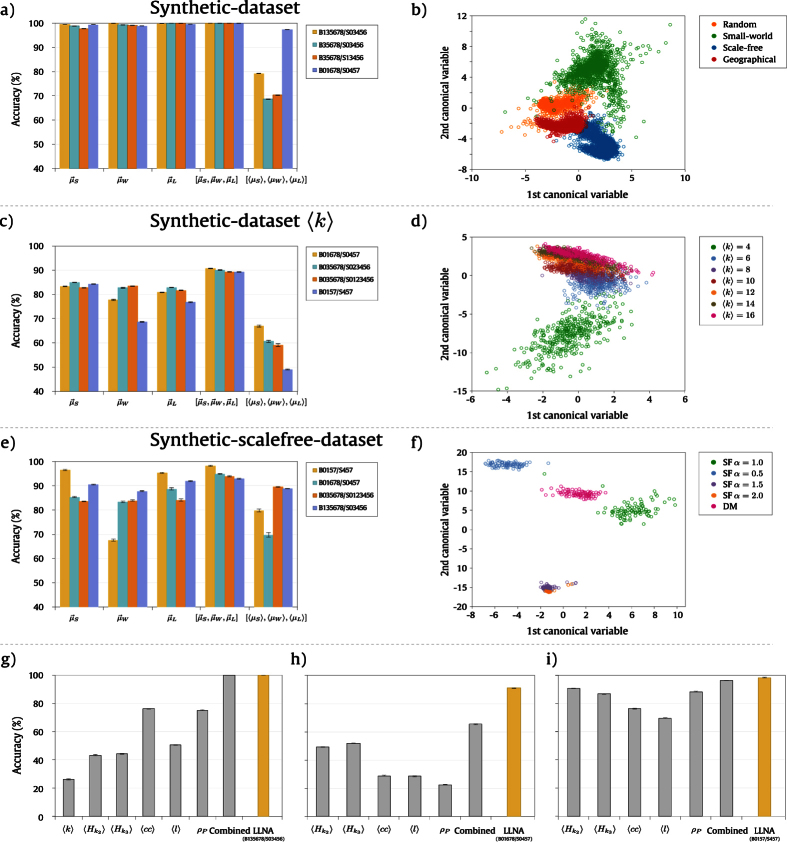
Synthetic network characterization with LLNA. (**a**) Accuracy (%) and standard deviation obtained in classifying network models: random, small-world, scale-free and geographical, using five different feature vectors and four Life-like rules. The vectors 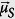, 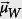 and 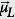 represent the distributions of the Shannon entropy, the word length and the Lempel-Ziv complexity, respectively. The vector 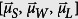 is composed by the combination of these distributions, and, [〈*μ*_*S*_〉, 〈*μ*_*W*_〉, 〈*μ*_*L*_〉] contains the average values of the same measurements. (**b**) Canonical analysis of the four network models using rule B135678/S03456 and 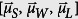 as feature vector. (**c**) Accuracy (%) obtained in classifying network models in combination with 〈*k*〉 as classes. (**d**) Canonical analysis of the 7 distinct values of 〈*k*〉 for the geographical network model using rule B01678/S0457 and 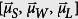. (**e**) Accuracy (%) obtained in classifying scale-free network models generated with linear and non linear preferential attachment. (**f**) Canonical analysis of (**e**) using rule B0157/S457 and 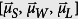. Plots (**g**), (**h**) and (**i**) present the comparison with structural measurements which are related to the plots presented in (**a**), (**c**) and (**e**), respectively. The following measurements were used: mean degree (〈*k*〉), average hierarchical degree of level 1 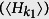, average hierarchical degree of level 2 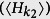, average clustering coefficient (〈*cc*〉), average path length (*l*) and degree Pearson correlation (*ρ*_*P*_). The best accuracy obtained by LLNA is highlighted in yellow.

Figure 5(a) presents the accuracy of four rules that are among the ten previously selected rules. We can observe high accuracy values for all the feature vectors except for the vector [〈*μ*_*S*_〉, 〈*μ*_*W*_〉, 〈*μ*_*L*_〉], which is composed by the average values of each measurement. The maximum accuracy obtained was 99.992 ± 0.002% for rule B135678/S03456 using the combination of the distributions 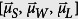. When analyzed separately, the distributions also provided high values of accuracy, especially the distribution of the Shannon entropy, 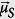. Figure 5(b) presents the canonical analysis for the *synthetic*-*dataset* using 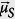 as attribute and rule B135678/S03456 as transition function. The canonical analysis is a regression analysis that provides a linear combination of the original attributes which maximizes the separation between the classes of interest^60^. Therefore, the first and the second canonical variables correspond to the eigenvectors with the highest eigenvalues of a matrix that quantifies the intra-class variation regarding the instances of the same class, and, another matrix which quantifies the inter-class variation among the classes. There is a clear separation among the four network models which corroborates the high accuracies obtained for the distributions as feature vectors. Additionally, Fig. 5(g) presents the comparison between the structural measurements and the best LLNA rule. Both approaches provided similar results (100% of accuracy considering the standard deviation). This can be explained by the fact that the networks used in this experiment were generated from classical theoretical models which present known properties that can be characterized by several measurements.

In another experiment, we evaluated the influence of the network mean degree, 〈*k*〉, in the spatio-temporal pattern. As shown in Fig. 2, different evolution patterns can be observed for the same network model given different values of 〈*k*〉. One question that can be raised is whether the spatio-temporal pattern for a given network model with specific 〈*k*〉 is unique. We performed this investigation considering now the combinations of 〈*k*〉 and the network models as classes. Therefore, we have a total of 28 classes, resulting from the combination of seven distinct values of 〈*k*〉, varying from 〈*k*〉 = 4 to 〈*k*〉 = 16, and four network models. This experiment was also performed with *synthetic*-*dataset*. The results regarding accuracy are shown in Fig. 5(c). The four rules highlighted in this figure are the ones that provided the highest accuracies among the ten selected rules. Using the same set of feature vectors, the maximum accuracy obtained was 90.76 ± 0.07% for rule B01678/S0457. This rate was also achieved using the combination of the distributions as attributes, and, when comparing the three distributions separately, we can see that 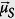 provided the highest accuracies individually for the selected rules. This result shows that we can distinguish the evolution pattern not only for the network models, but also for networks with distinct values of 〈*k*〉. The average measurements did not present a good performance as well as for the classification of the network models.

Figure 5(d) presents the canonical analysis regarding the 7 distinct values of 〈*k*〉 for the geographical network model using the feature vector and the rule that provided the best performance in Fig. 5(c). The confusion matrix for rule B01678/S0457 and the canonical analysis for the other network models are shown in Figs S2 and S3 of supplementary material, respectively. It can be observed that as the values of 〈*k*〉 increases the network topology tends to be highly connected, and, therefore, the error rate also increases and the discrimination among the classes becomes less clear, as shown in Fig. 5(d). These results are corroborated by measurements derived from the confusion matrix (see Table S6 of supplementary material). Considering, for instance, the values of the area under the curve in the ROC (*Receiver Operating Characteristic*) analysis, it can be observed that the AUC value decreases for larger values of 〈*k*〉 for all the network models. The performance measurements for all the 10 selected rules and for both experiments described so far can be found in Sections S6 and S7 of supplementary material. Finally, Fig. 5(h) presents the comparison between the structural measurements and the best LLNA rule (B01678/S0457). In this case, there was an improvement in the accuracy rate when using LLNA. The maximum accuracy obtained with the structural measurements as attributes was 65.2 ± 0.2% when combining five measurements in the same feature vector ([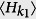, 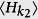, 〈*cc*〉, *l, ρ*_*P*_]). Therefore, the improvement in accuracy using LLNA was 25.5 ± 0.3%. In this analysis, we did not used 〈*k*〉 as attribute since we want to classify the network model in combination with 〈*k*〉.

In the third experiment with synthetic networks, we evaluated LLNA in the characterization of different scale-free models. We performed the classification of scale-free networks with both linear and non-linear preferential attachment: *α* = 0.5, 1.0, 1.5, 2.0. These networks were generated according to the well-known method proposed by Barabási & Albert^51^. We also considered another set of scale-free networks generated using the method proposed by Dorogovtsev & Mendes^52^. We used the *synthetic*-*scalefree*-*dataset* in this experiment, which contains instances of five distinct classes representing the different scale-free models. Similarly to the other experiments, the performance of each feature vector is shown in Fig. 5(e). The combination of the distributions also provided the highest accuracies for the *synthetic*-*scalefree*-*dataset* and the maximum accuracy obtained was 98.3 ± 0.2% by rule B0157/S457. The performance of the Shannon entropy and the Lempel-Ziv complexity distributions can also be highlighted. However, there is an heterogeneity regarding the performance of the feature vectors for each rule, e.g., the vector [〈*μ*_*S*_〉, 〈*μ*_*W*_〉, 〈*μ*_*L*_〉] performed very well for rule B035678/S0123456 (89.5 ± 0.2%). In contrast, the same feature vector provided accuracy of 70 ± 1% for rule B01678/S0457 (seesection S8 of supplementary material for quantitative results of all the ten selected rules). The obtained results indicate that even networks with similar topologies can provide specific temporal evolution, which may be used as signature vectors in a pattern recognition context. Figure 5(f) presents the canonical analysis for rule B0157/S457 and 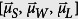 as feature vector. There is a clear separation among the three classes and an intersection between the scale-free models with *α* = 1.5 and *α* = 2.0. Finally, Fig. 5(i) presents the comparison with structural measurements for the *synthetic*-*scalefree*-*dataset*. LLNA also surpasses the accuracy obtained with the combination of structural measurements (96.20% ± 0.03) providing an improvement of 2.08 ± 0.25%.

### Pattern recognition in real-world applications

Three examples of LLNA in real-world applications are described in the next subsections. In all the experiments performed, we used the LLNA method to classify specific categories of each application. All the datasets used in the experiments were split into rule-selection and classification sets. The rule-selection set was used to perform the selection of the Life-like rules that could provide the best classification rates to discriminate classes of interest, whereas the classification set was used to evaluate the model. The details of the statistical approach used for the classification are described in Materials and Methods section.

#### Identifying organisms using metabolic networks

Metabolic networks describe the chemical reactions of the metabolic pathways that rule the transformations between chemical compounds through the action of enzymes. The aim of using LLNA is to characterize the metabolic networks of distinct organisms grouped by evolutionary classes. In this application, we investigated whether three distinct classes of organisms could be distinguished by the proposed method. The dataset used for this task was previously constructed by Jeong *et al*.^13^ and is publicly available^61^. This dataset contains 43 metabolic networks, which provides a description of the metabolic pathways of three types of organisms: archaea, bacteria and eukaryotes^13^. The original database was built based on the metabolic reactions found in the WIT database^62^. These metabolic networks were generated considering the educt-educt complexes and associated enzymes as representations of nodes and edges respectively.

**Figure 6:**
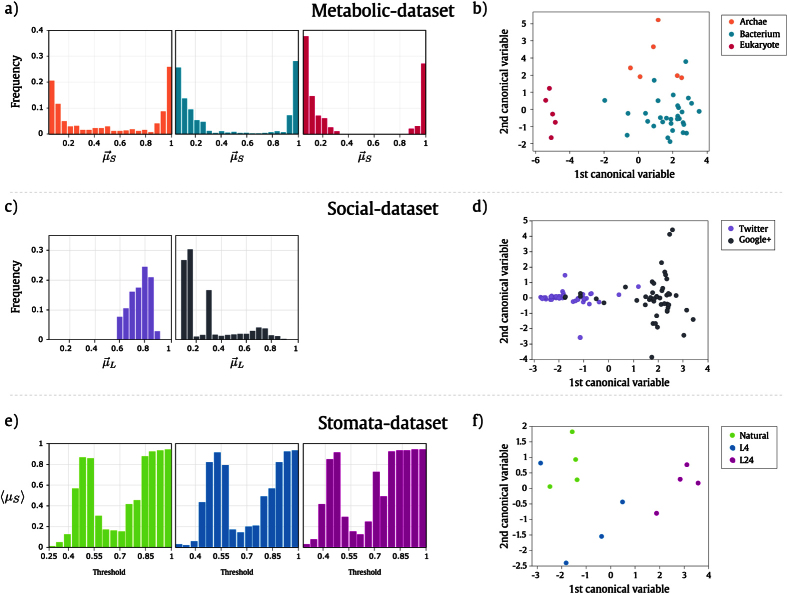
Characterization of real-world applications with LLNA. (**a**) Histogram of the Shannon entropy, 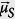, for three samples of each category of organisms. These histograms were generated using rule B05-S13568. (**b**) The corresponding canonical analysis of the *metabolic*-*dataset* highlighting the separation among the three classes. Similarly, (**c**) and (**d**) present the histogram of each class of the *social*-*dataset* and its canonical analysis. For this dataset the histograms were generated using the distribution of the Lempel-ziv complexity, 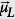, as feature vector and rule B0167-S248. Finally, (**e**) and (**f**) present the corresponding plots for the *stomata*-*dataset* using rule B12345/S04568. Specifically, (**e**) shows the average values of Shannon entropy 〈*μ*_*S*_〉 at each threshold *δ*_*T*_ for the different lighting conditions.

The first plot of Fig. 6(a) shows an example of the histograms representing the distributions of the Shannon entropy 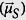 for one sample of each of the three classes. This histogram is used as the network descriptor and illustrates its behavior. These distributions were obtained through the spatio-temporal patterns resulting from the Life-like dynamics over the respective metabolic network. It can be observed distinct distributions for the three classes. For instance, the network of the “Eukaryote” class provided high frequency of low entropy values, which can be understood as the presence of more stable and/or oscillating patterns in the respective spatio-temporal diagram. The separation of the “Eukaryote” class is also clear in Fig. 6(b), which presents the canonical analysis for the *metabolic*-*dataset* using the same parameters and the same feature vector of the samples of Fig. 6(a). Both figures highlight the potential of the network descriptor to identify the classes of organisms.

**Figure 7:**
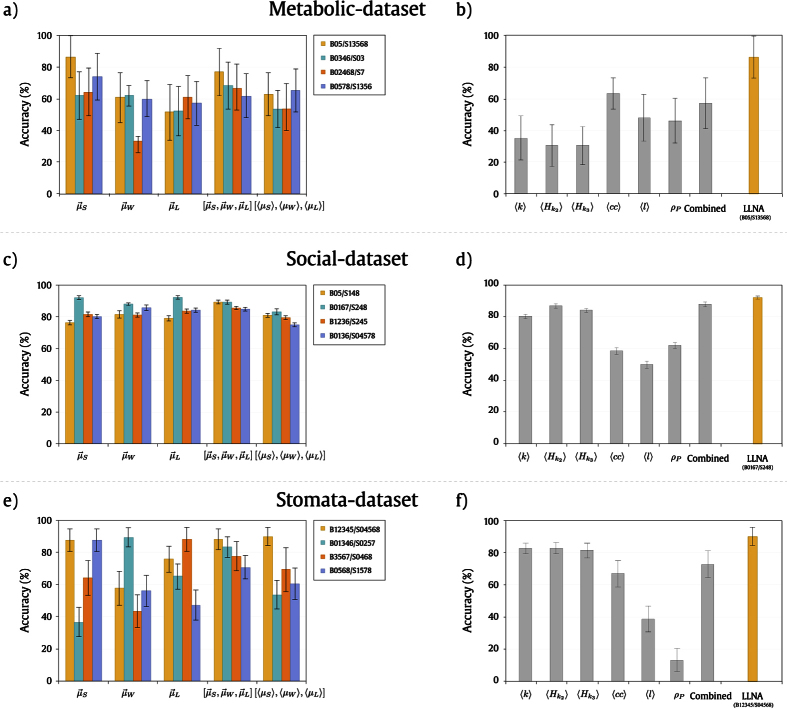
LLNA Validation: Plots (**a**), (**c**) and (**e**) present the classification accuracy and standard deviation obtained for the respective validation sets of each application for the best four rules and for all the feature vectors: the distribution of the Shannon entropy 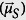, the distribution of the word length 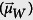, the distribution of the Lempel-ziv complexity 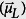, the combination of the previous three distributions (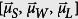) and average values of the same measurements ([〈*μ*_*S*_〉, 〈*μ*_*W*_〉, 〈*μ*_*L*_〉]). Plots (**b**), (**d**) and (**f**) show the classification accuracy (%) and standard deviation of the classes related to real-world applications using structural network measurements as feature vectors: mean degree (〈*k*〉), average hierarchical degree of level 1 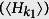, average hierarchical degree of level 2 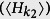, average clustering coefficient (〈*cc*〉), average path length (*l*) and degree Pearson correlation (*ρ*_*P*_) in comparison with the best accuracy obtained using LLNA (yellow).

The results regarding the performance of LLNA in the classification set for the metabolic networks are presented in Fig. 7(a). Specifically for the *metabolic*-*dataset* we used the re-sampling strategy, as described in the Materials and Methods section, as the number of samples per class is not uniform. The feature vector composed by the distribution of the Shannon entropy 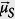 provided the highest accuracy value, 87 ± 13%, using rule B05/S13568. This percentage corresponds to the highest accuracy for the classification of the different domains of life. Additional performance measurements for this dataset can be found in section S9 of supplementary material. For instance, it is possible to observe from Table S10 that the descriptors obtained with LLNA could completely separate the “Eukaryote” class from the others. F-measure, MCC (Matthews Correlation Coefficient) and AUC (Area Under the Curve) using ROC analysis achieved 1.0 for this class. Finally, in Fig. 7(b) we can observe the comparison between the best accuracy obtained with LLNA and the accuracies obtained with different structural network measurements. In this case, LLNA provided an increase in the classification accuracy of 23 ± 23% when compared to the clustering coefficient attribute which provided the best accuracy among the network measurements, 64 ± 10%.

#### Identifying structural patterns in social networks

Social networks are examples of complex systems that have been studied for many decades using different theoretical approaches. More recently they have been used to illustrate several properties of complex networks. Online social networks offer a great variety of ways for social interactions and, in addition, supported by the technological advances, they can store a huge amount of data. Some of them present tools for sharing and grouping people in communities of specific topics. Different softwares for constructing social networks can bias the way people connect to each other, yielding this way, specific structures in the network. The goal of this experiment is use LLNA to identify the software tool used to create the social network. We used networks from the SNAP database^53^,^63^ in order to distinguish networks from Google+ and Twitter. In this context, LLNA was used to analyze different structural properties of both types of networks, which correspond to the classes of this application.

Figure 6(c) and (d) illustrate the differences regarding the spatio-temporal dynamics of each social network, Google+ and Twitter (see Materials and Methods for details about the *social*-*dataset*). The distributions presented in Fig. 6(c) illustrate that the descriptor can distinguish very well between the two classes. Notice that, the Twitter histogram presents the Lempel-Ziv values concentrated between 0.6 and 0.9, whereas the Google+ histogram presents the Lempel-Ziv values distributed across the histogram, with peaks in the beginning. The separation between both classes is clear in Fig. 6(d), which presents the canonical analysis for the *social*-*dataset*.

Regarding the classification performance of LLNA for this dataset, Fig. 7(c) presents the accuracies obtained for the different feature vectors and their combinations. The best accuracy value for distinguishing the evolution patterns of both social network tools, Google+ and Twitter, was obtained using the distribution of the Lempel-ziv complexity 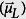, 92 ± 1%, and, rule B0167/S248. However, the feature vector 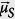 provided good accuracy as well for the same rule. When compared to the performance of the structural measurements (Fig. 7(d)), LLNA also surpasses the accuracy obtained when using the combination of these measurements as feature vector, 88 ± 2%. Therefore, we have an increase in the classification rate of 4 ± 3% for the *social*-*dataset* (see section S10 of supplementary material for additional performance measurements for the *social*-*dataset*).

#### Classifying stomata distribution patterns

Stomata distribution in leaves represents the phenotypic plasticity of plants, which is the ability to adapt their behavior to environmental conditions, such as light, temperature, amount of nutrients, among others. We used the LLNA method in order to characterize the phenotypic plasticity of the species *Tradescantia zebrina* to different light conditions regarding the distribution patterns formed by their stomata. We used an image dataset yielded by Florindo *et al*.^54^, which consists of six images for each lighting condition: sunlight (natural), 4 hours (L4) and 24 hours (L24) of artificial light. For modeling the stomata into a network, each stoma was segmented from the leaf image and its coordinates were assessed. For each image, a stomata network was modeled. The network represents the relationship of the centroids distance given a threshold radius *δ*_*T*_. As *δ*_*T*_ increases, more connections will be established between the centroids, and, therefore, the density of the network will be higher, producing a network dynamics that is used for image modeling. The construction of this network is detailed in Fig. S4 of the supplementary material. This approach for modeling images into networks was adapted from ref. 36. The main characteristic of this method is the concatenation of the network descriptors obtained at each value of *δ*_*T*_. We used 16 threshold values with *δ*_*i*_ = 0.25, incremented by 0.0625 until reaching a final threshold of *δ*_*f*_ = 1. Figure 6(e) presents the LLNA analysis of the *stomata*-*dataset*. We obtained the LLNA descriptors for the networks generated at each threshold *δ*_*T*_. The bar-plot shows the average values of the Shannon entropy 〈*μ*_*S*_〉 at each threshold *δ*_*T*_ for the different lighting conditions. The separation among the three classes is also highlighted in the canonical analysis shown in Fig. 6(f). We can see that the class “L24” is linearly separable from the others.

Figure 7(e) and (f) present the classification results for the *stomata*-*dataset*. The highest accuracy obtained for this dataset was 90 ± 6% using rule B12345/S04568 and [〈*μ*_*S*_〉, 〈*μ*_*W*_〉, 〈*μ*_*L*_〉] as feature vector, as reported in Fig. 7(e). The standard deviation for this dataset is higher due to the small number of instances for each class, and, there is also a higher heterogeneity regarding the behavior of the rules for the different feature vectors (see section S11 of supplementary material for the additional performance measurements for the *stomata*-*dataset*). When compared with structural measurements, LLNA provided an improvement in classification rate of 7 ± 9%. The best classification rate obtained using structural measurements was 83 ± 4%.

## Discussion

In this paper, we presented the Life-Like Network Automata (LLNA) method for pattern recognition in networks. LLNA uses a network as a tessellation of a CA and the feature extraction is based on the spatio-temporal patterns obtained through its evolution. We evaluated the performance of LLNA in two type of datasets: synthetic and real-world networks and we also performed the comparison of LLNA with structural network measurements obtained directly from the network topology when used as feature vectors.

The importance of the characterization of theoretical network models is related to the known properties of these models which may be useful in the comprehension of their spatio-temporal patterns. The first experiment considering four network models as classes (random, small-world, scale-free and geographical) provided a basic classification problem to evaluate the proposed method and LLNA could distinguish them with 99.992 ± 0.002% of accuracy. Additionally, we evaluated LLNA regarding its robustness to noise. We made structural changes in the network topology by randomly adding and removing edges according to a noise rate *ρ*_*N*_ (see Section S6.1 of supplementary material). The classification results obtained using this set of “noisy” networks also show a good performance of the proposed method, which evidences its robustness.

In the second experiment, we performed the classification considering the combinations of 〈*k*〉 and the network model as classes. Besides the heterogeneity of the dataset, which is composed by networks with different values of 〈*k*〉 and *N*, LLNA provided a good performance achieving 90.76 ± 0.07%. This experiment provided an analysis of the influence of the connectivity of the network in the spatio-temporal pattern. As the connectivity increases, the distinction between the patterns of network models is less accurate. We can see from the confusion matrix presented Fig. S2 of supplementary material that the error rate is higher for the classes representing networks with also higher 〈*k*〉. In the last experiment with synthetic networks, different scale-free models, with linear and non-linear preferential attachments, were distinguished using LLNA being 98.3 ± 0.2% the highest accuracy obtained. The *synthetic*-*scalefree*-*dataset* is composed by networks whose degree distributions are very similar. Nevertheless, LLNA could also capture the structural differences among the distinct classes of scale-free networks. Therefore, the preferential attachment parameter directly influences the spatio-temporal patterns.

For all experiments using synthetic networks, the analysis of the different feature vectors shows that the overall performance of the distributions 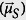, 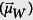 and, 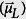 was higher when compared to the feature vector composed by the average values of the same measurements. The combination of the distributions of the selected measures (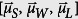) provided the best results when distinguishing the categories of interest in each experiment. Moreover, when analyzed separately, all the distributions were also very discriminative in many cases.

The accuracy provided by LLNA was compared with other structural network measurements. In the case of classifying network models, the performance of LLNA is as high as the performance obtained for a specific set of structural network measurements. Both approaches achieved maximum performance, which makes difficult to compare the methods. For the other two experiments (classification of 〈*k*〉 in combination with the network model and the classification of scale-free models), the classification task provided a better performance analysis, since both methods did not achieve the maximum performance. LLNA provided an improvement in accuracy of 25.5 ± 0.3% for the former, and 2.08 ± 0.25% for the latter, demonstrating to be a better discriminative method.

LLNA was evaluated in three real-world applications: identifying organisms using metabolic networks, identifying structural patterns in social networks, and, classifying stomata distribution patterns. Each application has a different scope allowing to analyze LLNA as a general tool for pattern recognition. Regarding the analysis of the metabolic networks, in the original study^13^, the authors showed that even organisms of distinct evolutionary classes present metabolic networks with similar topology. All of them have power-law degree distributions what characterizes them as scale-free networks. In addition, in this study, we have shown that pattern recognition algorithms can go a step further in terms of analyzing the network topology as they are able to find subtleties that can be used to distinguish networks within the same topological group, allowing the characterization of sub-categories of networks. The maximum accuracy obtained with LLNA was 87 ± 13% in contrast to 64 ± 10% using the clustering coefficient as feature vector. The two-class problem of distinguishing Twitter and Google+, and, the analysis of the stomata distribution patterns also demonstrated the feasibility of the proposed method as a pattern recognition tool. In the former, the different tools provided by each social network may influence the way people connect to each other resulting in structural differences between both social networks, although some properties such as the preferential connection of nodes and presence of hubs may exist in both of them. The maximum accuracy obtained with LLNA for this application was 92 ± 1% in contrast to 88 ± 2% using the combination of five structural measurements as attributes. In the latter application, the plant plasticity for different lighting conditions is reflected in the network of connections between the stomata centroids. In this case, the proposed method could capture the specific characteristics of the three classes of interest. For this application, the maximum accuracy obtained with LLNA was 90 ± 6% in contrast to 83 ± 4% using the hierarchical mean degree as feature vector. The performance of LLNA in the real-world applications was compared with the structural measurements. It provided a significant improvement in the correct classification rate as high as 23 ± 23% for the first, 4 ± 3% for the second and 7 ± 9% for the third application. The accuracy obtained using LLNA surpasses the accuracy obtained using traditional measurements as attributes, both individually and combined.

Besides the good performance of the proposed method, some characteristics of the method can be highlighted. LLNA is invariant to the size of the network. Networks with the same topology but with different sizes preserve the descriptor. This property is demonstrated in the Section S5 of supplementary material. The four synthetic networks (random, small-world, scale-free and geographical) were built with different number of nodes (500, 1000, 1500 and 2000) and the signature of each network model preserves its shape independently of the size. The method can also be extended to weighted and directed networks, which makes it suitable to a large number of applications which are based on di-graphs and that the weight of each link is important for the characterization. It was also demonstrated that the Life-like rule is the most influential parameter as the set of rules that provided the best classification rates are different for the distinct applications. In this study, we pointed out that among the 262144 rules of Life-like CA, there is a set of them that provides optimal solutions for a specific problem. Therefore, this set must be validated for each application. This issue can be explored in future studies by using optimization algorithms in order to reduce the time taken for the training phase. The proposed method outperformed structural measurements for the characterization in both synthetic and real-world networks, demonstrating to be a good choice for pattern recognition in networks. Therefore, potentially any pattern recognition application whose data is represented as a network can consider LLNA.

## Materials and Methods

### Generation of network models

We used the *igraph* library, a network analysis package, to support the implementation of some of the network models we used in this paper^64^. Random, small-world and scale-free networks of the Barabási & Albert model were generated using this library. The Dorogovtsev & Mendes scale-free networks and the geographical networks were implemented according to the proposed models^32^,^52^. Specifically, the geographical networks consist of nodes with specific spatial positions in contrast to networks defined in abstract spaces. Therefore, the connection between two nodes is given by the distance or geographical boundaries between them. We generated geographical networks by first defining the distribution of *N* nodes randomly in a bi-dimensional space. Then, the connections between the links were defined according to the following probability: 
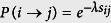
, where *s*_*ij*_ is the distance between nodes *i* and *j* and *λ* is the scale factor. The datasets of synthetic networks can be downloaded at: http://scg.ifsc.usp.br/LLNA.

### Datasets

In this section, we present the datasets used in order to evaluate our methodology, as well as the design of each experiment. We conducted experiments with two distinct types of networks. The first one consists of synthetic networks and the second one is composed by real-world networks. The first category of networks is organized into three datasets: *synthetic*-*dataset, rule*-*selection*-*dataset* and *synthetic*-*scalefree*-*dataset*. The second category is composed by the *metabolic*-*dataset* and the *rule*-*selection*-*metabolic*-*dataset*. Detailed information about these datasets is described next.

- *Synthetic*-*dataset* - composed of synthetic networks generated according to the following models: 1) random, with connection probability between two nodes of *p* = 〈*k*〉/*n*; 2) small-world, with rewiring probability of *p* = 0.1; 3) scale-free, with both linear and non-linear preferential attachments, and, 4) geographical. For each model, there are networks with the following values of 〈*k*〉: 4, 6, 8, 10, 12, 14, 16; and, the following values of *N*: 500, 1000, 1500 and 2000. We generated 100 networks for each of the 28 combinations of 〈*k*〉 − *N*. Therefore, the total number of networks in this dataset is 11200, and there are 2800 of each model;
- *Rule*-*selection*-*dataset* - composed of synthetic networks of the same four theoretical models used in *synthetic*-*dataset* and with the same generation parameters. However, in contrast, this dataset contains only networks with *N* = 500 nodes and 50 networks for each of the 7 combinations of 〈*k*〉 − *N*. The instances of this dataset are totally different from the *synthetic*-*dataset*;
- *Synthetic*-*scalefree*-*dataset* - composed of scale-free networks generated according to the models proposed by Barabási & Albert^51^ and Dorogovtsev & Mendes^52^. For the first model, we generated networks with both linear and non-linear preferential attachments (*α*): 0.5, 1.0, 1.5 and 2.0. Therefore, we have five classes in this dataset. The dataset contains 100 networks for each of these five classes with *N* = 1000 nodes and 〈*k*〉 = 8;
- *Metabolic*-*dataset* - The dataset of metabolic networks contains 43 samples which provide a description of the metabolic pathways of three types of organisms: 6 *archaea*, 32 *bacteria* and 5 *eukaryotes*^13^,^61^. This dataset was divided into two sets: rule-selection and classification. The first contains 2 randomly selected samples of each class, which were used to find the set of the best Life-like rules regarding their accuracy in distinguishing among the evolutionary classes. The second set consists of the remaining networks;
- *Social*-*dataset* This dataset contains networks from the SNAP (*Stanford Network Analysis Project*) platform^53^. We randomly selected 65 network samples for both Google+ and Twitter, which were divided into 15 samples of each one for the selection of the best Life-like rules and 50 for validation. All the social networks, also called “ego-networks” represents the social relationships or friends of a specific user (“ego”) that is not represented in the network;
- *Stomata*-*dataset* This dataset comprises digital binary images which represent the stomata distribution patterns of *Tradescantia zebrina* under three different illumination conditions: (i) sunlight (Natural), in which the plant is exposed to the sun light, (ii) 4 hours (L4) of artificial light, in which the plant is exposed to artificial light during 4 hours, and, (iii) 24 hours (L24) of artificial light, in which the plant is also exposed to artificial light, however during a larger period of 24 hours. The plants were expose to this conditions during 69 days. There are a total of 6 images for each condition, from which 2 were used for the rule-selection procedure and the other 4 for validation.

### Spatio-temporal measurements

The Shannon entropy (*μ*_*S*_)^65^ for node *i* is given by 
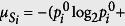
 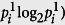, in which 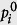 is the probability of zeros and 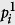 is the probability of ones in the time series. Word length (*μ*_*W*_) distribution considers the length of each word in the spatio-temporal series. A “word”, in this context, is a sequence of ones limited by zeros, e.g., *q* = (00**111**0**11**00), on which there is one word of length three and one word of length two. The Lempel-ziv complexity (*μ*_*L*_)^66^ is based on the number of different blocks of a sequence. The leftmost bit of a binary sequence *q* is the first block from which all other sub-sequences are constructed. Each new block is added to the dictionary. For example, the following binary sequence *q* = (01010101010101010101) has length *l* = 20 and is decomposed in seven *g* = 7 blocks as follows: “0|1|01|010|10|101|0101”. The Lempel-Ziv complexity is given by 
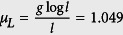
.

### Feature vectors

We selected a set of measurements in order to compose the feature vectors based on their discriminatory characteristics: 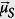, 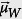 and 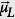. The first feature vector 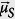 consist of the distribution of the Shannon entropy. The values of this measurement belong to the interval [0, 1]. In order to obtain 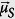, we calculated the Shannon entropy for each node, then, from these values we obtained a histogram by dividing the interval [0, 1] into 20 bins. Therefore, 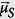 is composed by these 20 attributes, which represent the respective frequencies. The second feature vector 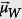 is composed by the word length distribution. In this context, a word is a sequence of ones limited by zeros, for instance, in the following sequence *q* = (00**111**0**11**00), we have one word of length three and one word of length two. The maximum word length is bound by the number of evolution steps, but due to the fact that the frequency of words with a length larger than 40 is very low, we considered only words smaller than this value. The histogram bin has length 2, so we also have 20 features for 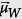. The last feature vector 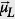 contains the Lempel-Ziv complexity distribution divided into 20 bins, this vector was normalized by the maximum value achieved among the group of samples. We also tested the average values for the same measures as attributes: average Shannon entropy, average word length and average Lempel-Ziv complexity: 〈*μ*_*S*_〉, 〈*μ*_*W*_〉, 〈*μ*_*L*_〉.

### Training and validation strategies

We used *n*-fold cross-validation strategy in all the experiments. This validation is a statistical method which consists of a generalized way to evaluate the prediction capacity of a model. Specifically in our case, we used cross-validation to evaluate LLNA regarding the accuracy in the classification performance for the pattern recognition applications. All datasets used were divided into a rule-selection dataset and a classification dataset. The cross-validation procedure was applied 100 times in both of them. Therefore, the standard deviation obtained is related to the variation in accuracy for each run of this procedure, since the assignment of the dataset instances to each fold is given randomly. k-NN (k - *Nearest Neighbors*) and SVM (*Support Vector Machines*) classifiers were used in the experiments. K-NN classifier is a simple voting algorithm in which the classes of the *k* nearest neighbors of a given instance are considered^67^. Whereas, SVM uses hyperplanes as decision boundaries of a classifier. The optimal hyperplane provides the maximal separation of the boundaries between two classes and is obtained by the solution of a quadratic optimization problem^68^. When the datasets did not present a uniform distribution of the classes, we used a random sub-sample strategy as the case for the *metabolic*-*dataset*. Specifically, we performed the classification step under a resampling k-fold strategy, with *k* = 3-folds using 100 random configurations for every group.

## Additional Information

**How to cite this article**: Miranda, G. H. B. *et al*. Exploring Spatio-temporal Dynamics of Cellular Automata for Pattern Recognition in Networks. *Sci. Rep.* **6**, 37329; doi: 10.1038/srep37329 (2016).

**Publisher’s note:** Springer Nature remains neutral with regard to jurisdictional claims in published maps and institutional affiliations.

## Supplementary Material

Supplementary Material

## Figures and Tables

**Figure 1:**
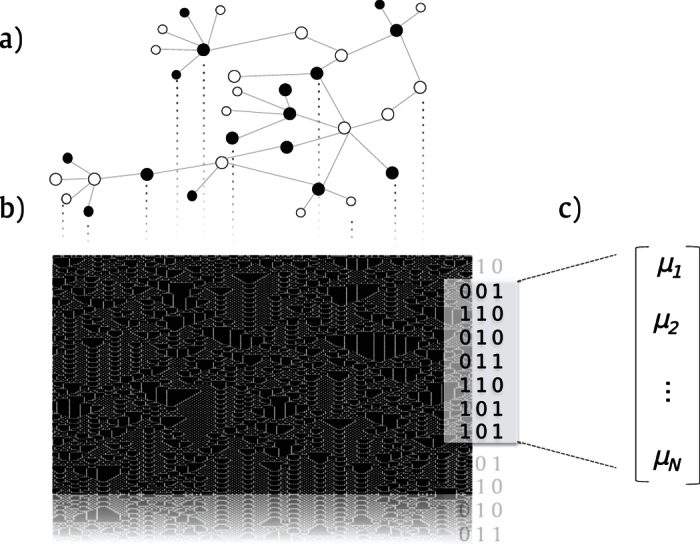
Pattern Recognition in networks using spatio-temporal patterns evolved by a cellular automata. (**a**) Modeling a binary cellular automata over the network topology. Black cells represent the nodes in the “on” state and white cells, the nodes in the “off” state. (**b**) Spatio-temporal diagram of the evolved automaton. Each column of the diagram represents the evolution of a single node and each row represents the configuration of the states at each time step. (**c**) Network descriptor represented by a vector of attributes obtained from the previous diagram.
