# Supplementary material for: Exploring Spatio-temporal Dynamics of Cellular Automata for Pattern Recognition in Networks
Source: Sci Rep. 2016 Nov 22;6:37329. doi: 10.1038/srep37329 (PMC5118793; doi:10.1038/srep37329)
Supplement: Supplementary Material [file srep37329-s1.pdf]

Supplementary Material

Exploring Spatio-temporal Dynamics of Cellular Automata for Pattern Recognition in Networks

Gisele Helena Barboni Miranda   Jeaneth Machicao   Odemir Martinez Bruno

Abstract

Network science is an interdisciplinary field which provides an integrative approach for the study of complex systems. In recent years, network modeling has been used for the study of emergent phenomena in many real-world applications. Pattern recognition in networks has been drawing attention to the importance of network characterization, which may lead to understanding the topological properties that are related to the network model. In this paper, the Life-Like Network Automata (LLNA) method is introduced, which was designed for pattern recognition in networks. LLNA uses the network topology as a tessellation of Cellular Automata (CA), whose dynamics produces a spatio-temporal pattern used to extract the feature vector for network characterization. The method was evaluated using synthetic and real-world networks. In the latter, three pattern recognition applications were used: (i) identifying organisms from distinct domains of life through their metabolic networks, (ii) identifying online social networks and (iii) classifying stomata distribution patterns varying according to different lighting conditions. LLNA was compared to structural measurements and surpasses them in real-world applications, achieving improvement in the classification rate as high as 23%, 4% and 7% respectively. Therefore, the proposed method is a good choice for pattern recognition applications using networks and demonstrates potential for general applicability.

S1 Life-Like Cellular Automata

Cellular Automata (CA) are discrete mathematical models that can be used to describe the interactions between the elements (cells) of a system. Due to their abstract and dynamical nature, CA have shown to be suitable for modeling a vast amount of applications. At the same time, one interesting characteristic that is drawing attention of CA is the temporal pattern obtained from its evolution. Some of these patterns are characterized by emergent behaviors which arises from simple deterministic rules and may lead to the formation of complex patterns [10].

Formally, CA consist of a tessellation defined on a n-dimensional space and their fundamental units are called cells. For each cell $i = 1, 2, ..., N$ a state s_i is defined in time t, which is also represented by $s(c_i, t)$. CA are discrete in time and space, and their states are updated according to deterministic rules, also called transition rules, ϕ. These rules depend on the current state of cell i and on the states of its neighborhood. A CA is defined by the quintuple [1]: $\mathcal{C} = \langle \mathcal{T}, S, s_0, N, \phi \rangle$, on which:

- $\mathcal{T}$, is an infinite tessellation on a n-dimensional Euclidean space $\mathbb{R}^n$, that consists of cells $c_i, i \in \mathbb{N}$;
- S, a set of n states, such that $S \in \mathbb{N}$;
- s_0, $\mathcal{T} \to S$ assigns to every cell c_i an initial state, *i.e.*, $s(c_i, 0) = s_0(ci)$. Notice that σ_s is the probability of having a state s. For instance, $\sigma_1 = 50\%$ represents an uniform distribution of the initial states having ones as state cell.
- N, a function that defines a neighborhood of each cell: $N : \mathcal{T} \to \cup_{p=1}^{\infty} \mathcal{T}^p$. This function maps each cell c_i to a finite sequence $N(c_i) = (c_{ij})_{j=1}^{|N(c_i)|}$ that consists in $|N(c_i)|$ distinct cells;
- ϕ, an output function, such that $\phi : \mathbb{N} \to S$. This function determines the state of cell c_i in time t, *i.e.,* $s(c_i, t)$;

A tessellation $\mathcal{T}$ may be composed of cells on manifold formats, regular or irregular. The cells are assumed to be homogeneous, *i.e.,* they present the same properties and follow the same transition rules [8]. The state of each cell depends only on what is happening in its local neighborhood, that is why CA have parallel architecture. Interesting patterns and particular properties were found on the famous CA Game of Life, or simply Life, introduced by Martin Gardner in the 70s [6]. Life is defined over an orthogonal bi-dimensional grid with a set of binary states: alive or dead, where each cell interacts with its eight neighbors (Moore neighborhood).

Life-like CA are a family of cellular automata inspired by the rules of Life, which are defined on the same topology and the same neighborhood, however, the set of transition functions vary. For example, the original Life rule is defined by the following notation: B3/S23, *i.e.,* a cell is born if there are 3 neighbors alive and a cell survives if at least 2 or 3 neighbors are alive. B and S are strings containing values on the interval $[0 - 8]$. Since each cell can have a maximum of 8 neighbors, there are a total of 2^{18} possible transition rules when combining the conditions for birth (B) and survival (S). Some of the transition rules Bx/Sy were deeply investigated regarding the patterns formed by their time evolution even acquiring names. Moreover, each of these rules presents particular properties that have been employed for specific purposes.

S2 Measurements extracted from Life-Like Network Automata

This section details three different measurements used to extract features from the spatio-temporal patterns obtained trough the evolution of the Life-Like Network Automata.

Shannon entropy (μ_S)

The Shannon entropy [9] for a given node i is defined by $\mu_{S_i} = -(p_i^0 \log_2 p_i^0 + p_i^1 \log_2 p_i^1)$, where p_i^0 is the probability of having zeros in the time series and p_i^1 is the probability of having ones. This measure quantifies how homogeneous is the evolution pattern and is normalized between $[0, 1]$. Spatio-temporal series containing only zeros or ones present $\mu_S = 0$. Whereas, oscillating patterns tend to present higher Shannon entropy.

Word length (μ_W)

Tthe Word length (μ_W) describes how homogeneous is the signal regarding the length of the "words". In this context, a "word" is a sequence of ones limited by zeros, for instance, in the following sequence $q = (00\mathbf{111}0\mathbf{11}00)$, we have one word of length three and one word of length two. The number of evolution steps t is the largest possible word length, which is obtained when a node starts the evolution alive and never changes its state.

Lempel-ziv complexity (μ_L)

The Lempel-ziv complexity is derived from the data compression algorithm proposed by Lempel&Ziv [7]. The Lempel-Ziv complexity measurement, (μ_L), is one of the derivations proposed by the referred authors which is based in a dictionary of sub-sequences. This algorithm counts the number of different blocks a sequence contains. The leftmost bit of a binary sequence q defines the first block. From this bit, one moves rightward bit by bit, until a sub-sequence formed from the previous block up to the current position has not appeared before, then, this new block is added to the dictionary. For example, the following binary sequence $q = (01010101010101010101)$ of length $l = 20$ is decomposed into $g = 7$ minimum blocks "0|1|01|010|10|101|0101", so the Lempel-Ziv complexity is $\mu_L = \frac{g \log l}{l} = 1.049$.

S3 Structural network measurements

Given the adjacency matrix A, where $a_{ij} = 1$, if i is connected to j and $a_{ij} = 0$, otherwise, then, the number of neighbors or degree of node i is defined as: $k_i = \sum_{j=1}^{N} a_{ij}$, where N is the total number of nodes. Therefore, we have the following measurements [3]:

- Average Degree – $\langle k \rangle$:

  $$\langle k \rangle = \frac{1}{N} \sum_i k_i$$

- Average Hierarchical Degree (level 2) – $\langle H_{k_2} \rangle$: the hierarchical degree of level 2, H_{k_2}, of a given node i, $H_{k_2}(i)$, is the sum of the degrees of its neighbors. Therefore, the average hierarchical degree of level 2, is given by:

  $$\langle H_{k_2} \rangle = \frac{1}{N} \sum_i H_{k_2}(i)$$

- Average Hierarchical Degree (level 3) – $\langle H_{k_3} \rangle$: the hierarchical degree of level 3, H_{k_3}, of a given node i, $H_{k_3}(i)$, is the sum of the degrees of the neighbors of its neighbors. Therefore, the average hierarchical degree of level 3, is given by:

  $$\langle H_{k_3} \rangle = \frac{1}{N} \sum_i H_{k_3}(i)$$

- Average Clustering Coefficient ($\langle cc \rangle$): the clustering coefficient of node i, cc_i, measures the probability of two vertices j and k being connected to each other since both are connected to node i and is calculated by $cc_i = 2e_i/k_i(k_i - 1)$. The average clustering coefficient is given by:

  $$\langle cc \rangle = \frac{1}{N} \sum_i cc_i$$

- Average Path Length (l): the average path length is the average length of the shortest paths between any two nodes, i and j, of the network.

  $$l = \frac{1}{N(N-1)} \sum_{i \neq j} d_{ij}$$

- Degree Pearson Correlation (ρ):

  $$\rho = \frac{(1/M) \sum_{j>i} k_i k_j a_{ij} - [(1/M) \sum_{j>i} (1/2)(k_i + k_j) a_{ij}]^2}{(1/M) \sum_{j>i} (1/2)(k_i^2 + k_j^2) a_{ij} - [(1/M) \sum_{j>i} (1/2)(k_i + k_j) a_{ij}]^2}$$

S4 Performance measurements

The experiments performed in this study were evaluated using a set of performance measurements which are described in this section. These measurements were calculated for each experiment in order to evaluate the results obtained with LLNA in each specific application. The terminology derives from the confusion matrix: True Positive (TP), True Negative (TN), False Positive (FP), type I error, and, False Negative (FN), type II error.

- Accuracy (ACC): the rate of correctly classified instances

  $$ACC = \frac{TP + TN}{TP + FP + TN + FN}$$

- True Positive Rate (TPR): the rate of true positives (percentage of positive correctly classified instances for a given class)

  $$TPR = \frac{TP}{TP + FN}$$

- False Positive Rate (FPR): the rate of false positives (percentage of negative incorrectly classified instances for a given class)

  $$FPR = \frac{FP}{FP + TN}$$

- F1-*score* is a measure derived from the combination of precision and recall:

  $$F1 = \frac{2TP}{2TP + FP + FN}$$

- Matthews Correlation Coefficient (MCC)

  $$\frac{TP \times TN - FP \times FN}{\sqrt{(TP + FP)(TP + FN)(TN + FP)(TN + FN)}}$$

- Area Under a ROC Curve (AUC):

  $$AUC = \frac{R_1}{n_1 n_2} - \frac{n_1(n_1 + 1)}{2n_1 n_2}$$

  where n_1, n_2 is the sample size for sample 1 and 2, respectively, and R_1 is the sum of the ranks in sample 1. Following the MannWhitney U statistic test.

S5 Analysis and Selection of Parameters: influence of the number of Nodes N

This section introduces the analysis of the number of network nodes, N, regarding its influence on the spatio-temporal pattern obtained by applying LLNA. Fig. S1 presents the histograms of the Shannon entropy, $\vec{\mu}_S$, using rule B01678/S0457 for the four network models studied in this paper: random, small-world, scale-free and geographical. We can see that distinctly from the number of evolution steps, t, and from the distribution of initial alive population, σ, the parameter N does not influence $\vec{\mu}_S$. The histograms for distinct values of N are very similar given the same network model.

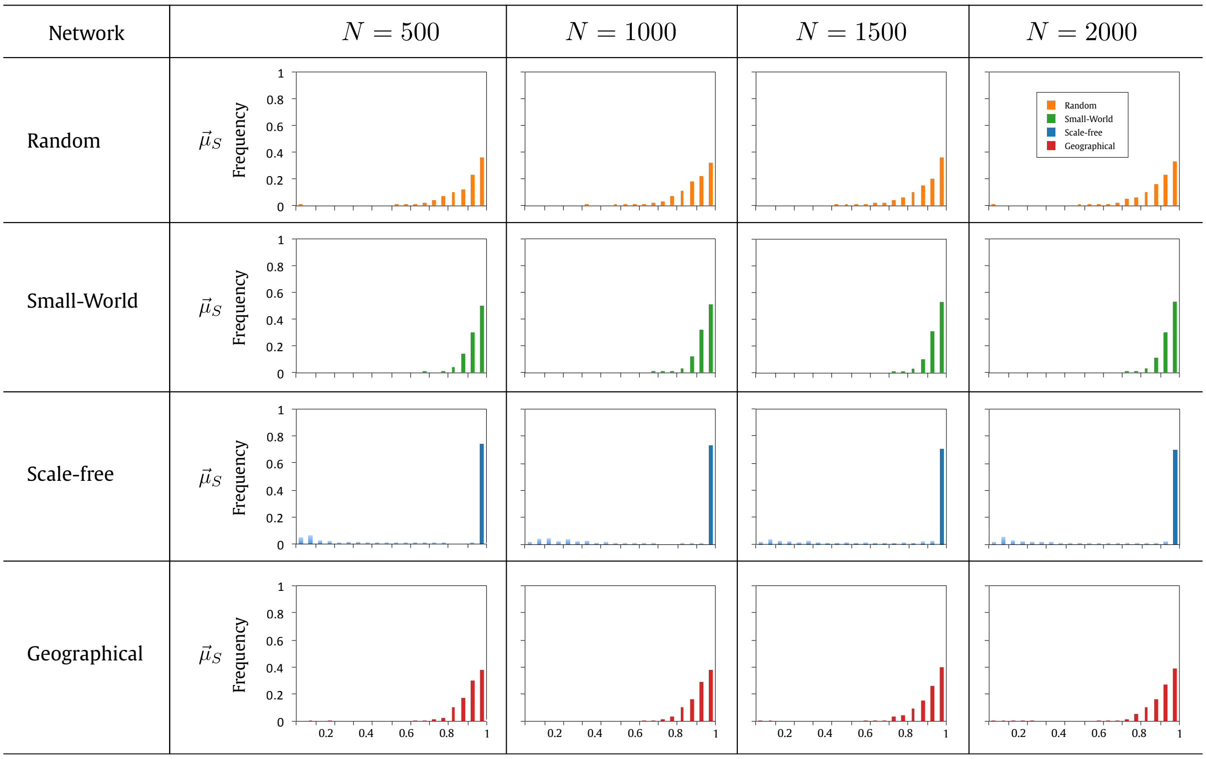

Figure S1: Histograms of the Shannon entropy distribution $\vec{\mu}_S$ for each of the four network models (random, small-world, scale-free and geographical) for different values of the number of nodes $N = 500, 1000, 1500, 2000$. All these networks have $\langle k \rangle = 8$ and were evolved during $t = 350$ time steps.

S6 Classifying network models

Table S1 presents accuracy rates in classifying network models for the 10 selected Life-like rules. Columns $\vec{\mu}_S$, $\vec{\mu}_W$ and $\vec{\mu}_L$ show the correct classification rates for each rule when using Shannon entropy, word length and Lempel-Ziv distributions as attributes, respectively. The results when combining these distributions are shown in the fifth column of the same table. This combination leads to a maximum accuracy of 99.992 ±0.002% for rule B135678/S03456. Finally, the last column shows the accuracies when using the average values of the same measures as attributes: $\langle\mu_S\rangle$, $\langle\mu_W\rangle$ and $\langle\mu_L\rangle$. Table S2 presents the performance measurements for the same experiment.

Table S1: Correct classification rate (%) obtained using different measures and their combinations as attributes to classify the following network models: random, small-world, scale-free and geographical. The networks were built with $N = 500, 1000, \ldots 2000$ and mean degree varying from $\langle k \rangle = 4, 6 \ldots 16$.

Rules	$\vec{\mu}_S$	$\vec{\mu}_W$	$\vec{\mu}_L$	$[\vec{\mu}_S, \vec{\mu}_W, \vec{\mu}_L]$	$[\langle\mu_S\rangle, \langle\mu_W\rangle, \langle\mu_L\rangle]$
B01678/S0457	99.37 (±0.01)	98.87 (±0.01)	99.55 (±0.01)	99.946 (±0.003)	97.34 (±0.02)
B035678/S023456	98.91 (±0.02)	99.33 (±0.02)	98.96 (±0.02)	99.89 (±0.01)	84.31 (±0.03)
B1245678/S3456	97.40 (±0.03)	96.09 (±0.01)	97.67 (±0.02)	99.80 (±0.01)	95.65 (±0.01)
B0157/S457	97.19 (±0.04)	87.74 (±0.03)	94.60 (±0.03)	98.67 (±0.04)	82.60 (±0.03)
B35678/S03456	98.77 (±0.02)	99.26 (±0.03)	99.900 (±0.004)	99.98 (±0.01)	68.61 (±0.05)
B1245678/S03456	96.24 (±0.04)	96.67 (±0.03)	95.57 (±0.02)	99.91 (±0.01)	95.65 (±0.01)
B35678/S13456	97.70 (±0.05)	99.03 (±0.03)	99.92 (±0.01)	99.94 (±0.01)	70.28 (±0.08)
B01245678/S3456	97.73 (±0.03)	97.93 (±0.02)	91.63 (±0.04)	99.68 (±0.01)	94.57 (±0.05)
B035678/S0123456	98.39 (±0.02)	98.93 (±0.02)	98.34 (±0.03)	99.79 (±0.01)	84.67 (±0.05)
**B135678/S03456**	99.47 (±0.02)	99.912 (±0.003)	99.76 (±0.01)	**99.992 (±0.002)**	79.30 (±0.06)

Table S2: Performance measurements corresponding to the best accuracy as shown in Table S1.

TP Rate	FP Rate	F-Measure	MCC	ROC Area	Class
1	0	1	1	1	RAN
1	0	1	1	1	SW
1	0	1	1	1	SF
1	0	1	1	1	GEO
**1** ±0	**0** ±0	**1** ±0	**1** ±0	**1** ±0	**Avg**

S6.1 Robustness to noise

The robustness of the LLNA method was evaluated regarding noise tolerance. The network topology was modified by the removal and the addition of edges according to a noise rate ρ_N. As this rate increases, more structural changes are performed on the network topology. A noise rate of 20% ($\rho_N = 20\%$) indicates the addition of 10% of edges, regarding the total number of edges, and, the removal of 10% of the existing edges. Both set of edges are randomly selected. Table S3 shows the correct classification rate of the four network models when considering different values of ρ_N and using SVM classifier. The feature vector composed by the combination of the distributions, $[\vec{\mu}_S, \vec{\mu}_W, \vec{\mu}_L]$, provided the best accuracy. In general, we can observe that the increase of ρ_N does not influence the accuracy obtained for each rule since the changes of accuracy values were very small.

Table S3: Analysis of the robustness of the proposed method: Network classification accuracy (%) given different values of ρ_N, and, also without noise.

Rules	Without Noise	$\rho_N = 10\%$	$\rho_N = 20\%$	$\rho_N = 30\%$
B01678/S0457	99.946 (±0.003)	99.85 (±0.01)	99.70 (±0.01)	99.84 (±0.01)
B035678/S023456	99.89 (±0.01)	99.90 (±0.01)	99.79 (±0.01)	99.95 (±0.01)
B1245678/S3456	99.80 (±0.01)	99.79 (±0.02)	99.51 (±0.02)	99.73 (±0.01)
B0157/S457	98.67 (±0.04)	99.44 (±0.02)	99.73 (±0.02)	99.85 (±0.01)
B35678/S03456	99.98 (±0.01)	99.96 (±0.01)	99.91 (±0.01)	99.990 (±0.003)
B1245678/S03456	99.91 (±0.01)	99.80 (±0.01)	99.61 (±0.02)	99.68 (±0.02)
B35678/S13456	99.94 (±0.01)	99.94 (±0.01)	99.93 (±0.01)	99.97 (±0.01)
B01245678/S3456	99.68 (±0.01)	99.54 (±0.01)	99.48 (±0.02)	99.62 (±0.02)
B035678/S0123456	99.79 (±0.01)	99.86 (±0.01)	99.84 (±0.01)	99.93 (±0.01)
B135678/S03456	99.992 (±0.002)	99.983 (±0.004)	99.97 (±0.01)	99.95 (±0.01)

S6.2 Comparison with structural measurements

The comparison between the performance of LLNA and the performance using structural measurements as feature vectors is shown in. Table S4. The structural measurements were also evaluated regarding their robustness to noise. The best individual scores were obtained by the average clustering coefficient ($\langle cc \rangle$) and by the Pearson degree correlation (ρ_P). The combination of the highlighted measurements reached 100.00% of accuracy, even in the presence of noise. LLNA achieved nearly the same accuracy with rule B135678/S03456. Regarding the network robustness to noise, some traditional measurements have shown to be more affected by the variation of ρ_N, *e.g*, $\langle l \rangle$ and ρ_P, whereas the others are more stable.

Table S4: Correct classification rate (%) obtained using the traditional network measures as attributes to classify the network models, and, the noise tolerance analysis using the same measures.

Measure	Without Noise	$\rho_N = 10\%$	$\rho_N = 20\%$	$\rho_N = 30\%$
$\langle k \rangle$	26.2 (±0.4)	26.0 (±0.5)	26.1 (±0.4)	26.2 (±0.4)
$\langle H_{k_2} \rangle$	43.4 (±0.4)	43.5 (±0.5)	43.5 (±0.5)	44.2 (±0.5)
$\langle H_{k_3} \rangle$	44.3 (±0.2)	44.0 (±0.3)	43.5 (±0.4)	43.1 (±0.4)
$\langle cc \rangle$	76.11 (±0.02)	75.64 (±0.02)	75.92 (±0.02)	75.79 (±0.02)
$\langle l \rangle$	50.46 (±0.05)	43.82 (±0.04)	42.7 (±0.2)	41.6 (±0.3)
ρ_P	75.2 (±0.2)	83.72 (±0.03)	85.87 (±0.03)	85.90 (±0.03)
combined	100.0 (±0.0)	100.0 (±0.0)	100.0 (±0.0)	100.0 (±0.0)
LLNA (B135678/S03456)	99.992 (±0.002)	99.983 (±0.004)	99.97 (±0.01)	99.95 (±0.01)

S7 Classifying network models in combination with $\langle k \rangle$

Table S5 presents the accuracy in classifying the network models in combination with the mean degree $\langle k \rangle$ as classes, Table S6 shows the performance measurements for the best rule (B01678/S0457), and, Table S7 presents the comparison of LLNA with structural measurements. The performance of LLNA in this case surpasses the performance of the structural measurements with an improvement of 25.54 ± 0.31%. The mean degree influence in the classification of the network models can also be analyzed in Figs S2 and S3. The first presents the confusion matrix for rule B01678/S0457 and the combination of distributions as feature vector $[\vec{\mu}_S, \vec{\mu}_W, \vec{\mu}_L]$ and the second presents the canonical analysis for the four network models. From both figures we can see that not all $\langle k \rangle$ values can be discriminated. For instance, for the scale-free model it is possible to distinguish networks with mean degree 4 (red), 6 (yellow) and 8 (green), while the other classes are closer to each other. A similar behavior is observed for the geographical model. Random and small-world models also have a set of $\langle k \rangle$ values that are also distinguishable from the others.

Table S5: Correct classification rate (%) obtained using different measures and their combinations as attributes to classify the four network models using their $\langle k \rangle$ as classes.

Rules	$\vec{\mu}_S$	$\vec{\mu}_W$	$\vec{\mu}_L$	$[\vec{\mu}_S, \vec{\mu}_W, \vec{\mu}_L]$	$[\langle \mu_S \rangle, \langle \mu_W \rangle, \langle \mu_L \rangle]$
**B01678/S0457**	83.35 (± 0.09)	77.8 (± 0.3)	80.87 (± 0.07)	**90.76 (± 0.07)**	67.0 (± 0.4)
B035678/S023456	84.97 (± 0.06)	82.8 (± 0.2)	82.91 (± 0.07)	90.12 (± 0.07)	60.6 (± 0.5)
B1245678/S3456	80.26 (± 0.08)	73.9 (± 0.1)	80.66 (± 0.05)	86.76 (± 0.08)	54.4 (± 0.4)
B0157/S457	84.33 (± 0.07)	68.7 (± 0.1)	76.8 (± 0.2)	89.34 (± 0.09)	49.1 (± 0.2)
B35678/S03456	77.9 (± 0.1)	77.5 (± 0.1)	75.3 (± 0.1)	86.76 (± 0.09)	62.3 (± 0.5)
B1245678/S03456	80.23 (± 0.08)	72.90 (± 0.08)	80.68 (± 0.06)	86.41 (± 0.07)	54.3 (± 0.4)
B35678/S13456	76.74 (± 0.09)	79.3 (± 0.1)	74.75 (± 0.08)	87.2 (± 0.1)	51.7 (± 0.4)
B01245678/S3456	77.80 (± 0.08)	75.10 (± 0.08)	79.65 (± 0.05)	86.37 (± 0.08)	45.7 (± 0.3)
B035678/S0123456	82.8 (± 0.1)	83.48 (± 0.09)	81.75 (± 0.07)	89.39 (± 0.08)	59.1 (± 0.6)
B135678/S03456	83.82 (± 0.09)	80.8 (± 0.2)	83.48 (± 0.05)	88.93 (± 0.07)	60.9 (± 0.3)

Table S6: Performance measurements corresponding to the best accuracy as shown in Table S5.

**TP Rate**	**FP Rate**	**F-Measure**	**MCC**	**ROC Area**	**Class**
1	0	0.996	0.996	1	RAN-k4
1	0	1	1	1	RAN-k6
0.983	0	0.991	0.991	1	RAN-k8
0.963	0.002	0.949	0.948	0.998	RAN-k10
0.868	0.006	0.858	0.853	0.993	RAN-k12
0.743	0.01	0.737	0.727	0.987	RAN-k14
0.81	0.005	0.83	0.824	0.995	RAN-k16
1	0	1	1	1	SW-k4
1	0	1	1	1	SW-k6
0.988	0.002	0.971	0.97	0.999	SW-k8
1	0	1	1	1	SW-k10
0.95	0.001	0.966	0.965	0.999	SW-k12
0.968	0	0.979	0.978	0.998	SW-k14
0.993	0.001	0.983	0.982	0.999	SW-k16
1	0	1	1	1	SF-k4
0.985	0.001	0.981	0.981	0.999	SF-k6
0.97	0.001	0.977	0.977	0.999	SF-k8
0.94	0.002	0.94	0.938	0.998	SF-k10
0.893	0.004	0.898	0.894	0.994	SF-k12
0.705	0.018	0.647	0.635	0.985	SF-k14
0.553	0.011	0.601	0.589	0.984	SF-k16
0.993	0	0.996	0.996	1	GEO-k4
1	0	1	1	1	GEO-k6
0.975	0.001	0.976	0.975	0.999	GEO-k8
0.883	0.004	0.881	0.877	0.994	GEO-k10
0.75	0.009	0.75	0.741	0.988	GEO-k12
0.715	0.012	0.698	0.686	0.985	GEO-k14
0.815	0.005	0.836	0.83	0.995	GEO-k16
0.9 $\pm$0.1	0.003$\pm$0.004	0.9 $\pm$0.1	0.9 $\pm$0.1	0.996$\pm$0.004	**Avg$\pm$ sd**

Table S7: Accuracy (%) of the *synthetic-dataset* regarding the classification of network models and $\langle k \rangle$ using structural network measurements as feature vectors. Moreover, the best accuracy of LLNA using rule B05/S13568 and $\vec{\mu}_S$ as feature vector.

	Accuracy
$\langle H_{k_2} \rangle$	49.1 ($\pm$0.1)
$\langle H_{k_3} \rangle$	51.83 ($\pm$0.08)
$\langle cc \rangle$	28.8 ($\pm$0.3)
$\langle l \rangle$	28.3 ($\pm$0.3)
ρ_P	22.4 ($\pm$0.2)
combined	65.2 ($\pm$0.2)
LLNA (B01678/S0457)	90.76 ($\pm$0.07)

Predicted Class

Real Class		Random							Small-world							Scale-free							Geographical						
	k	4	6	8	10	12	14	16	4	6	8	10	12	14	16	4	6	8	10	12	14	16	4	6	8	10	12	14	16
Random	4	100																											
	6		100																										
	8			99	1																								
	10				96	4																							
	12				6	86	8																						
	14					12	74	14																					
	16						18	82																					
Small-world	4								100																				
	6									100																			
	8										99		1																
	10											100																	
	12										4		95	1															
	14												1	96	3														
	16													1	99														
Scale-free	4															100													
	6																98	2											
	8																2	97	1										
	10																		94	6									
	12																		5	89	5	1							
	14																			2	69	29							
	16																			2	43	55							
Geographical	4	1																					99						
	6																							100					
	8																								98	2			
	10																								2	88	10		
	12																									9	75	16	
	14																										15	71	14
	16																											18	82

Figure S2: Confusion matrix for rule B01678/S0457 using the following feature vector $[\vec{\mu}_S, \vec{\mu}_W, \vec{\mu}_L]$, which corresponds to the best accuracy as shown in Table S5. Each cell represents a percentage (%) of the correct predicted class, where filled cells represents nonzero elements.

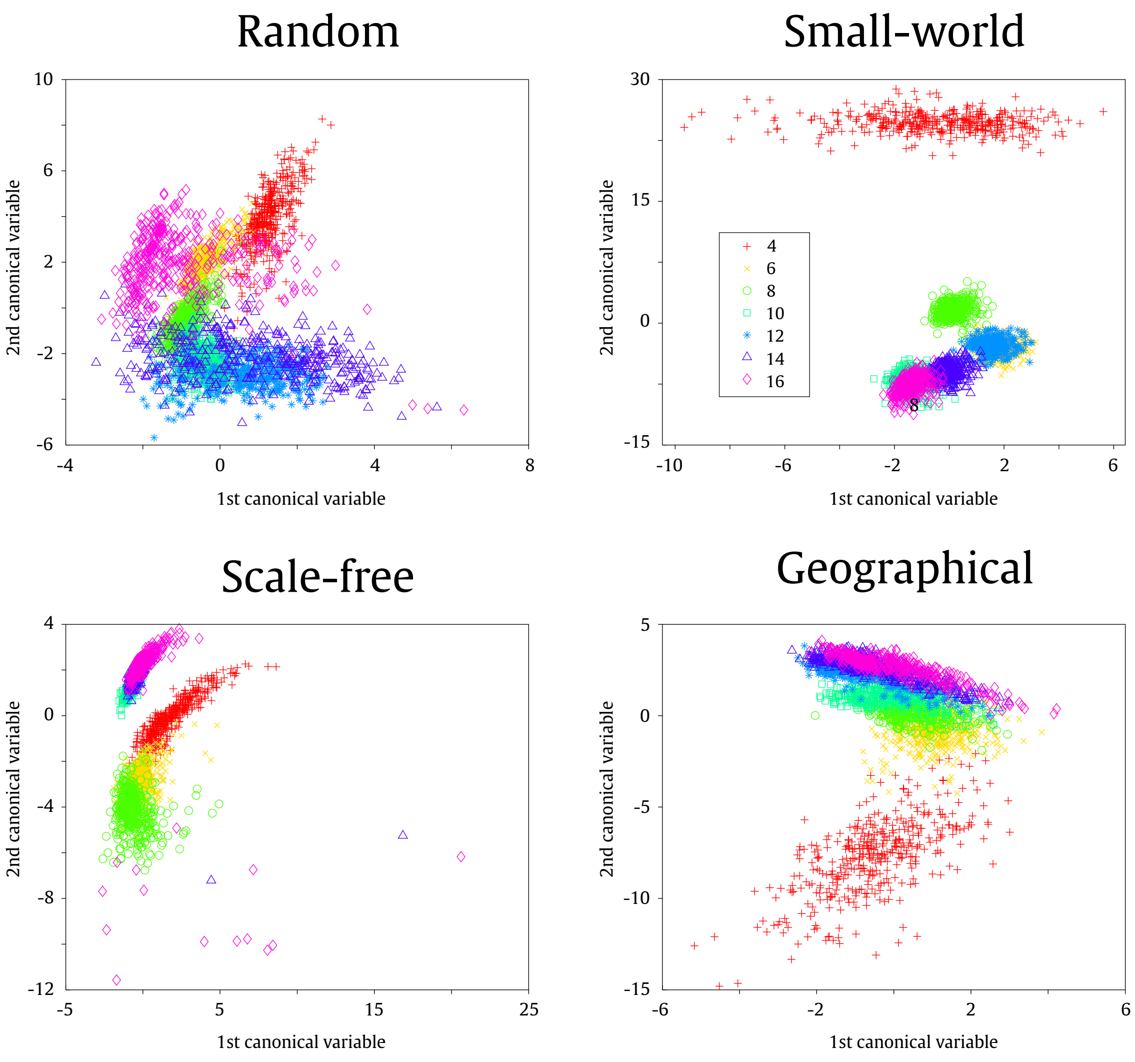

Figure S3: Canonical analysis for each network model considering distinct values of $\langle k \rangle$ as classes. For each model, there are networks with the following values of $\langle k \rangle$: 4, 6, 8, 10, 12, 14, 16 (*synthetic-dataset*). This analysis was performed using rule B01678/S0457, which provided the highest accuracy as shown in Table S5.

S8 Classifying scale-free models

Table S8 presents the accuracy obtained in classifying different scale-free models, with linear and non linear preferential attachment ($\alpha = \{0.5, 1.0, 1.5, 2.0\}$) using the Barabsi&Albert model [2], and, networks generated according to the model of Dorogovtsev&Mendes [4]. Table S9 shows the performance measurements for the best rule (B0157/S457). Finally, Table S10 presents the comparison with structural measurements. The improvement in accuracy provided by LLNA was 2.08±0.25% in this experiment.

Table S8: Classification accuracy (%) of five scale-free models using different measurements and their combinations as attributes

Rules	$\vec{\mu}_S$	$\vec{\mu}_W$	$\vec{\mu}_L$	$[\vec{\mu}_S, \vec{\mu}_W, \vec{\mu}_L]$	$[\langle\mu_S\rangle, \langle\mu_W\rangle, \langle\mu_L\rangle]$
B01678/S0457	85.4 (± 0.2)	83.4 (± 0.3)	88.8 (± 0.4)	94.9 (± 0.2)	70 (± 1)
B035678/S023456	82.77 (± 0.08)	75.8 (± 0.5)	83.7 (± 0.4)	85.8 (± 0.5)	79.200 (± 0.001)
B1245678/S3456	81.5 (± 0.4)	79.5 (± 0.1)	86.9 (± 0.2)	87.0 (± 0.2)	79.800 (± 0.001)
**B0157/S457**	96.5 (± 0.2)	67.6 (± 0.4)	95.3 (± 0.2)	**98.3 (± 0.2)**	79.8 (± 0.6)
B35678/S03456	89.0 (± 0.2)	86.2 (± 0.2)	81.0 (± 0.8)	88.4 (± 0.3)	83.8 (± 0.2)
B1245678/S03456	82.4 (± 0.3)	81.0 (± 0.3)	87.61 (± 0.08)	87.5 (± 0.1)	79.800 (± 0.001)
B35678/S13456	84.2 (± 0.2)	91.81 (± 0.03)	81 (± 1)	92.6 (± 0.1)	86.5 (± 0.3)
B01245678/S3456	79.9 (± 0.2)	79.7 (± 0.2)	85.7 (± 0.1)	85.5 (± 0.1)	79.80 (± 0.02)
B035678/S0123456	83.6 (± 0.1)	84.0 (± 0.3)	84.1 (± 0.5)	94.0 (± 0.2)	89.5 (± 0.2)
B135678/S03456	90.5 (± 0.2)	87.8 (± 0.2)	92.0 (± 0.2)	92.9 (± 0.2)	88.82 (± 0.06

Table S9: Performance measurements corresponding to the best accuracy of Table S8.

**TP Rate**	**FP Rate**	**F-Measure**	**MCC**	**ROC Area**	**Class**
0.98	0	0.99	0.987	0.994	BA$\alpha = 1.0$
1	0.003	0.995	0.994	0.999	DM
0.95	0.003	0.969	0.962	0.991	BA$\alpha = 2.0$
1	0.003	0.995	0.994	0.999	BA$\alpha = 0.5$
0.99	0.013	0.971	0.963	0.992	BA$\alpha = 1.5$
1.0±0.0	0.004±0.003	1.0 ±0.0	1.0±0.0	0.995±0.003	**Avg± sd**

Table S10: Accuracy (%) for the *synthetic-scalefree-dataset* using structural network measurements as feature vectors and the best accuracy of LLNA using rule B05/S13568 and $\vec{\mu}_S$ as feature vector

	Accuracy
$\langle H_{k_2}\rangle$	90.6 (±0.1)
$\langle H_{k_3}\rangle$	86.7 (±0.2)
$\langle cc\rangle$	76.3 (±0.2)
$\langle l\rangle$	69.6 (±0.2)
ρ_P	88.4 (±0.3)
combined	96.20 (±0.04)
LLNA (B0157/S457)	98.3 (±0.2)

S9 Identifying organisms using metabolic networks

Table S11 presents the accuracy results regarding the *metabolic-dataset* for the 10 selected rules. Table S12 shows the performance measurements for the classification of the best case reported for this using rule B05/S13568 and $\vec{\mu}_S$ as feature vector. Moreover, Table S13 presents the comparison of LLNA with other structural measurements for the same dataset.

Table S11: Validation of the classification accuracy (%) of the metabolic networks using different network measures and their combinations as attributes

Rules	$\vec{\mu}_S$	$\vec{\mu}_W$	$\vec{\mu}_L$	$[\vec{\mu}_S, \vec{\mu}_W, \vec{\mu}_L]$	$[\langle\mu_S\rangle, \langle\mu_W\rangle, \langle\mu_L\rangle]$
**B05-S13568**	**87 (± 13)**	61 (± 16)	52 (± 18)	77 (± 15)	63 (± 14)
B0346-S03	62 (± 15)	62 (± 7)	52 (± 16)	68 (± 15)	53 (± 12)
B02468-S7	64 (± 16)	33 (± 0)	61 (± 14)	67 (± 15)	54 (± 16)
B0578-S1356	74 (± 15)	60 (± 11)	57 (± 14)	62 (± 14)	65 (± 14)
B01237-S13	68 (± 14)	33 (± 0)	55 (± 16)	62 (± 16)	39 (± 16)
B01247-S2347	61 (± 16)	56 (± 10)	32 (± 15)	57 (± 18)	35 (± 15)
B3468-S12346	39 (± 16)	49 (± 12)	69 (± 16)	54 (± 15)	28 (± 16)
B2345678-S367	27 (± 15)	67 (± 2)	38 (± 17)	48 (± 17)	49 (± 16)
B23468-S45	40 (± 16)	37 (± 14)	42 (± 14)	44 (± 20)	33 (± 18)
B01678-S013578	40 (± 16)	31 (± 16)	33 (± 16)	37 (± 18)	71 (± 16)

Table S12: Performance measurements corresponding to the rule that provided the best accuracy rate of Table S12

**TP Rate**	**FP Rate**	**F-Measure**	**MCC**	**ROC-AUC**	**Class**
1.0	0.000	1.0	1.0	1.0	*Eukaryote*
0.667	0.000	0.800	0.756	0.833	*Bacterium*
1.0	0.167	0.857	0.791	0.917	*Archae*
0.9 ±0.1	0.06±0.07	0.9±0.1	0.8±0.1	0.92±0.06	Avg± sd

Table S13: Correct classification rate obtained (%) for the *metabolic-dataset* using structural network measures as feature vectors. The last row presents the results obtained for LLNA using B05/S13568 and $\vec{\mu}_S$ as feature vector

	Accuracy
$\langle k \rangle$	35 (± 14)
$\langle H_{k_2} \rangle$	30 (± 13)
$\langle H_{k_3} \rangle$	30 (± 12)
$\langle cc \rangle$	64 (± 10)
$\langle l \rangle$	48 (± 15)
ρ_P	46 (± 14)
combined	57 (± 16)
LLNA (B05/S13568)	87 (± 13)

S10 Identifying structural patterns in social network

Table S14 presents the accuracy results regarding the *social-dataset* for the best 10 select rules. Table S15 shows the true-positive rate, false-positive rate, precision and recall, F-measure, MCC and ROC area for the classification of the best case reported on the latter experiment using rule B0167/S248 and $\vec{\mu}_L$ as descriptor. Moreover, Table S16 presents the comparison with traditional measurements for the same dataset.

Table S14: Validation of the classification accuracy (%) of the social networks dataset using different network measures and their combinations as attributes.

Rules	$\vec{\mu}_S$	$\vec{\mu}_W$	$\vec{\mu}_L$	$[\vec{\mu}_S, \vec{\mu}_W, \vec{\mu}_L]$	$[\langle\mu_S\rangle, \langle\mu_W\rangle, \langle\mu_L\rangle]$
**B0167-S248**	**92 (± 1)**	88 (± 1)	**92 (± 1)**	89 (± 1)	83 (± 2)
B05-S148	76 (± 1)	82 (± 2)	79 (± 2)	89 (± 1)	81 (± 1)
B06-S148	79 (± 2)	79 (± 2)	75 (± 2)	78 (± 2)	81 (± 2)
B256-S3467	89 (± 1)	78 (± 2)	87 (± 1)	80 (± 1)	80 (± 1)
B05-S1458	84 (± 1)	80 (± 1)	81 (± 2)	82 (± 1)	80 (± 2)
B1236-S245	82 (± 2)	81 (± 1)	84 (± 1)	86 (± 1)	79 (± 1)
B456-S01237	80 (± 2)	65 (± 2)	78 (± 2)	82 (± 2)	79 (± 1)
B01267-S035678	78 (± 2)	75 (± 2)	69 (± 2)	70 (± 2)	79 (± 2)
B012368-S01245	85 (± 1)	82 (± 1)	76 (± 1)	78 (± 1)	79 (± 2)
B08-S1467	84 (± 1)	78 (± 3)	78 (± 2)	75 (± 1)	79 (± 2)

Table S15: Performance measurements corresponding to the rule that provided the best accuracy rate of Table S14

**TP Rate**	**FP Rate**	**F-Measure**	**MCC**	**ROC-AUC**	**Class**
0.900	0.040	0.928	0.862	0.930	Google+
0.960	0.100	0.932	0.862	0.930	Twitter
0.93 ±0.03	0.070±0.03	0.930±0.002	0.8±0.0	0.9±0.0	Avg± sd

Table S16: Correct classification rate obtained (%) for the *social-dataset* using structural network measures as feature vectors. The last row presents the results obtained for LLNA using B0167/S248 and $\vec{\mu}_L$ as feature vector

	Accuracy
$\langle k\rangle$	80 (± 1)
$\langle H_{k_2}\rangle$	87 (± 1)
$\langle H_{k_3}\rangle$	84 (± 1)
$\langle H_{k_3}\rangle$	59 (± 2)
$\langle cc\rangle$	59 (± 2)
$\langle l\rangle$	50 (± 2)
ρ_P	62 (± 2)
combined	88 (± 2)
LLNA (B0167/S248)	92 (± 1)

S11 Classifying stomata distribution patterns

Fig. S4 depicts the construction of the network of interactions for the *stomata-dataset*. Fig. S4-a) is the original microscopic image of a leaf from *Tradescantia zebrina*. Fig. S4-b) represents the stomata centroids, which were manually segmented. Finally, Fig. S4-c) shows the resulting network given the threshold $\delta_T = 0.9062$.

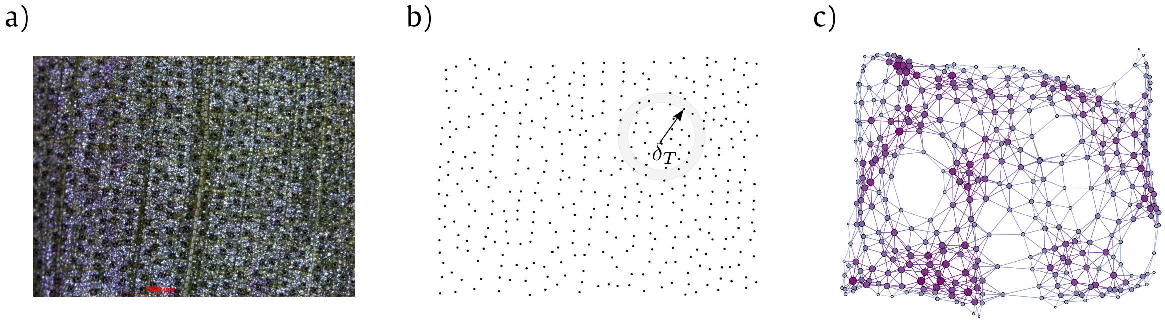

Figure S4: a) Microscopic image of a *Tradescantia zebrina* leaf, and, b) the respective stomata distribution under 4h of artificial lighting [5]. c) Network obtained using $\delta_T = 0.9062$.

Table S17 presents the accuracy results regarding the *stomata-dataset* for the best 10 select rules. Table S18 shows the true-positive rate, false-positive rate, precision and recall, F-measure, MCC and ROC area for the classification of the best case reported on the latter experiment using rule B12345/S04568 and $[\langle\mu_S\rangle, \langle\mu_W\rangle, \langle\mu_L\rangle]$ as descriptor. Moreover, Table S19 presents the comparison with traditional measurements for the same dataset.

Table S17: Validation of the classification accuracy (%) of the stomata networks dataset using different network measures and their combinations as attributes.

Rules	$\vec{\mu}_S$	$\vec{\mu}_W$	$\vec{\mu}_L$	$[\vec{\mu}_S, \vec{\mu}_W, \vec{\mu}_L]$	$[\langle\mu_S\rangle, \langle\mu_W\rangle, \langle\mu_L\rangle]$
**B12345-S04568**	87 (± 7)	58 (± 11)	76 (± 8)	88 (± 7)	**90 (± 6)**
B01346-S0257	37 (± 9)	90 (± 6)	65 (± 8)	83 (± 6)	54 (± 9)
B3567-S0468	64 (± 11)	44 (± 10)	88 (± 8)	78 (± 9)	69 (± 14)
B0568-S1578	88 (± 7)	56 (± 10)	47 (± 9)	71 (± 7)	61 (± 10)
B125678-S012345	68 (± 13)	54 (± 9)	87 (± 7)	66 (± 7)	84 (± 9)
B34-S024568	88 (± 7)	40 (± 7)	84 (± 10)	85 (± 7)	68 (± 9)
B35678-S012578	60 (± 9)	53 (± 10)	75 (± 9)	68 (± 10)	86 (± 8)
B02345-S01456	70 (± 9)	84 (± 8)	75 (± 8)	81 (± 9)	65 (± 8)
B056-S0348	66 (± 6)	85 (± 9)	66 (± 10)	67 (± 7)	63 (± 10)
B245-S0234568	77 (± 8)	34 (± 7)	87 (± 7)	78 (± 9)	63 (± 9)

Table S18: Performance measurements corresponding to the rule that provided the best accuracy rate of Table S17

**TP Rate**	**FP Rate**	**F-Measure**	**MCC**	**ROC-AUC**	**Class**
1.000	0.125	0.889	0.837	0.938	Natural
0.750	0.000	0.857	0.816	0.875	L4
1.000	0.000	1.000	1.000	1.000	L24
0.9±0.1	0.04±0.06	0.9 ±0.1	0.9 ±0.1	0.94 ±0.04	Avg± sd

Table S19: Correct classification rate obtained (%) for the *stomata-dataset* using structural network measures as feature vectors. The last row presents the results obtained for LLNA using rule B12345/S04568 and $[\langle \mu_S \rangle, \langle \mu_W \rangle, \langle \mu_L \rangle]$ as feature vector

	Accuracy
$\langle k \rangle$	83 (±3)
$\langle H_{k_2} \rangle$	83 (±4)
$\langle H_{k_3} \rangle$	81 (±5)
$\langle cc \rangle$	67 (±8)
$\langle l \rangle$	39 (±8)
ρ_P	13 (±7)
combined	74 (±8)
LLNA (B12345/S04568)	90 (± 6)

References

[1] Jan M Baetens and Bernard De Baets. Cellular automata on irregular tessellations. *Dynamical Systems*, 27(4):411–430, 2012.

[2] Albert-László Barabási and Réka Albert. Emergence of scaling in random networks. *Science*, 286(5439):509–512, 1999.

[3] L da F Costa, Francisco A Rodrigues, Gonzalo Travieso, and Paulino Ribeiro Villas Boas. Characterization of complex networks: A survey of measurements. *Advances in Physics*, 56(1):167–242, 2007.

[4] Sergey N Dorogovtsev and Jose FF Mendes. Evolution of networks. *Advances in physics*, 51(4):1079–1187, 2002.

[5] Joao Batista Florindo, Gabriel Landini, Humberto Almeida Filho, and Odemir Martinez Bruno. Analysis of stomata distribution patterns for quantification of the foliar plasticity of tradescantia zebrina. In *Journal of Physics: Conference Series*, volume 633, page 012113. IOP Publishing, 2015.

[6] Martin Gardner. Mathematical games: The fantastic combinations of john conway's new solitaire game "life". *Scientific American*, 223(4):120–123, 1970.

[7] Abraham Lempel and Jacob Ziv. On the complexity of finite sequences. *IEEE Trans. Inf. Theor.*, 22(1):75–81, 1976.

[8] Cosma Rohilla Shalizi. Methods and techniques of complex systems science: An overview. In *Complex systems science in biomedicine*, pages 33–114. Springer, 2006.

[9] C.E. Shannon. A mathematical theory of communication. *Bell System Technical Journal, The*, 27(3):379–423, 1948.

[10] Stephen Wolfram. Complex systems theory. In *Emerging Syntheses in Science: Proceedings of the Founding Workshops of the Santa Fe Institute (Addison-Wesley, 1988)*, 1985.
